# Analyzing the horizontal orientation of the crustal stress adjacent to plate boundaries

**DOI:** 10.1038/s41598-023-42433-2

**Published:** 2023-09-20

**Authors:** Tobias Stephan, Eva Enkelmann, Uwe Kroner

**Affiliations:** 1 Department of Geoscience, University of Calgary, Calgary, AB T2N 1N4 Canada; 2 TU Bergakademie Freiberg, Institute for Geology, 09599 Freiberg, Germany; 3 Department of Geology, Lakehead University, Thunder Bay, ON P7B 5E1 Canada

**Keywords:** Structural geology, Tectonics, Geophysics, Geodynamics

## Abstract

The spatial analysis of horizontal stress orientation is important to study stress sources and understand tectonics and the deformation of the lithosphere. Additional to the stress sources, the geometry of stress fields depends on the underlying coordinate reference system, which causes spatial distortions that bias the analysis and interpretation of stresses. The bias can be avoided when the stress field is decomposed and transformed into the reference frame of its first-order stress source. We present a modified and extended theory based on the empirical link between the orientation of first-order stresses and the trajectories of lateral plate boundary forces. This link is applied to analyze the orientation of horizontal stresses, their patterns, and tectonic structures from the perspective of their first-order source or cause. By using only parameters for the relative motion between two neighboring plates, we model the first-order orientation of the maximum horizontal stress that statistically fits the orientation of $\ge$80% of the global stress data adjacent to plate boundaries. Considerable deviations of the observed stress from the predicted first-order stress direction can reveal the geometry of second-order stresses and confine areas where other stress sources dominate. The model’s simple assumptions, independence from the sample size, potential application to regional to global scale analysis, and compatibility with other spatial interpolation algorithms make it a powerful method for analyzing stress fields. For immediate use, the presented method is implemented in the free and open-source software package tectonicr, which is written in the computer language R.

## Introduction

Observations of stress are fundamental Earth science data that are used to study forces that act in the lithosphere in order to understand deformation or stability of the crust and the underlying mantle. The orientation of the stress tensors allows for identifying and quantifying the relative contribution of different sources of stress. These sources range from plate scale to meter scale and include plate tectonic forces, forces originating from mechanical discontinuities, lithologic boundaries, intrusions, topography, and man-made structures (e.g. Refs.^1–4^). Tectonic forces are the most dominant source and plate boundary forces confine the kinematics of plate motion and the dynamics of plate deformation, which can result in major differences between intraplate and plate boundary deformation zones. These differences reflect the laterally and vertically heterogeneous deformation pattern of the crust due to its composition, mechanical properties, and tectonic setting. Understanding the stress sources, therefore, requires a thorough analysis of the stress field, which can then be used for stress predictions. Many methods exist for stress analysis, stress interpolation or smoothing, like inverse distance interpolation^5–7^ or nearest-neighbor regression^8^ that are both based on circular statistics assuming von Mises distributions (cf.^9^). Furthermore, non-linear smoothing for multi-dimensional data^10^, finite-element modeling^11^, damped inversion of focal mechanisms^12^, Bayesian formulation^13^, and cluster analysis^14,15^ have been used to interpolate stress fields. All these methods assume that the stress orientation can be averaged from the orientation of neighboring data samples. However, the geometry of a stress field largely depends on two main factors that are usually underestimated or ignored:

(i) In-situ stress measurements can be the result of a complex assemblage of an unknown number of sources with different magnitudes and orientations of underlying forces. On larger scales, these unknown contributions lead to unique and complex geometries of the stress field. Thus, the analysis requires either in-depth knowledge of the stress source(s) or a decomposition of the stress field into its constituents.
(ii) Patterns of large-scale stresses are affected by the Earth’s curvature. Thus, the geometries of stress fields are strongly dependent on the chosen coordinate reference system (CRS) and projection. Each combination of a CRS and a projection yields different geometries. For that reason, spherical mathematics and the choice of the CRS are equally important for characterizing the stress-field geometry. This is particularly crucial for differentiating between a uniform and a heterogeneous stress field.

This study demonstrates that the empirical link between the orientation of horizontal stresses at plate boundaries and the direction of relative plate motion provides a heuristic approach to analyzing stress fields. This stress-analysis approach accounts for the aforementioned factors. Our approach to determining stress orientations is based on the “theory of intraplate tectonics” proposed by Wdowinski^16^. We modify and extend his data analysis technique by decomposing and transforming the field into the reference frame of its first-order stress source and using modern spherical mathematics. We demonstrate and test our data-analysis technique using the stress fields of the regions of the San Andreas Faul–Gulf of California, Central Asia, the North Atlantic Ridge–Iceland, and the global stress field using the World Stress Map Database Release 2016 (WSM2016)^17^.

## Theoretical background

### The influence of the coordinate reference system on the stress-field geometry

**Figure 1:**
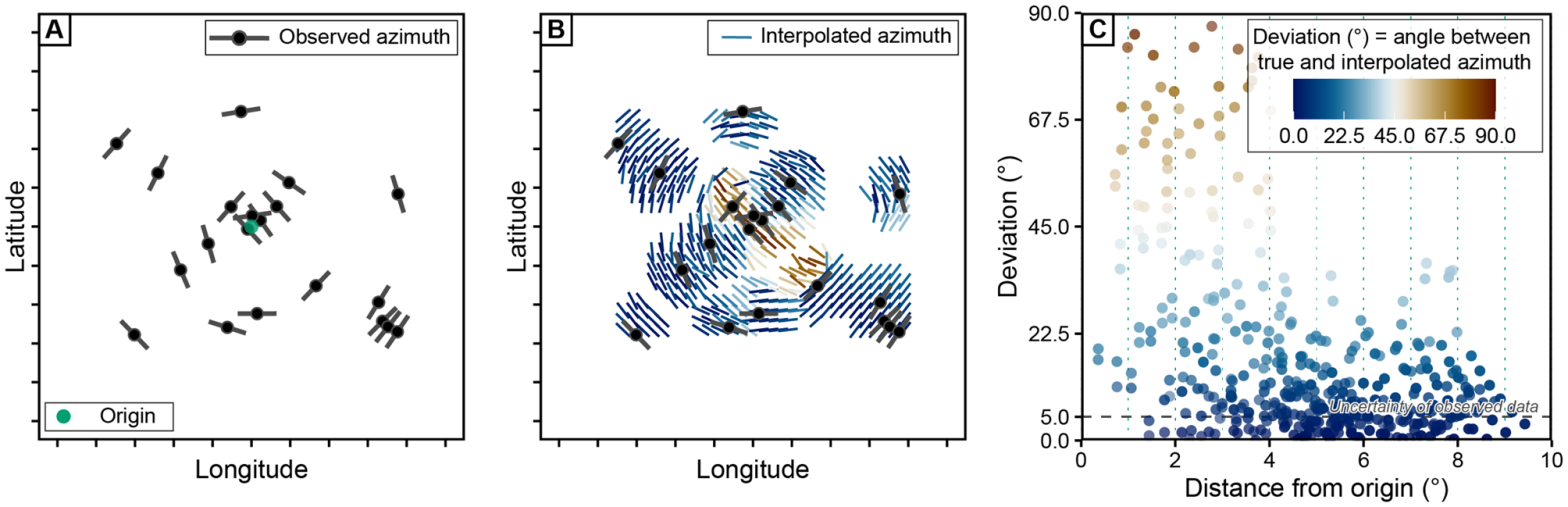
Stress-field predictions for a concentric stress field in a geographical coordinate reference system. (**A**) Synthetically concentric stress field presented on a map view (geographical coordinate reference system, Mercator projection) is centered at the origin of the concentric stress field. The orientation of the maximum horizontal stress ($\sigma _\text {Hmax}$) of the “observed” points is derived from a randomly sampled concentric point set. A 5$^{\circ }$ scatter around the true orientation is assigned to the orientation. (**B**) Spatial stress interpolation in a geographical reference system with the $\sigma _\text {Hmax}$ orientation measured as the deviation from the North Pole. Note the large deviation close to the origin of the stress field. (**C**) Deviation of the interpolated orientation from the true orientation plotted against the distance from the origin. The maximum deviation of the interpolated orientation exceeds the uncertainty of the observed orientation and can be as large as 90$^{\circ }$. Interpolation for the $\sigma _\text {Hmax}$ orientation are based on the “stress2grid” algorithm^6,21^ which calculates the circular mean orientation within a variable search radius (parameters: grid size = 0.5$^{\circ }$, search radius = 250–850 km).

The orientation of stress and strain is conventionally expressed by its azimuthal deviation from the geographic North Pole. Depending on the underlying geographic projection, the geometrical or mathematical analysis may produce spurious patterns of the stress (or strain) field as they are affected by different angle or area distortions. Figure 1 demonstrates how a synthetic stress field can be distorted and how this affects stress-field interpolation using circular statistical parameters to estimate the average stress orientation. This stress field comprises concentrically distributed data points with concentrically oriented maximum horizontal stress ($\sigma _\text {Hmax}$). Such a stress field may represent, e.g., a normal fault system circling an impact crater^18^, a volcanic caldera^19^, or the stress field above a diapiric structure^20^.

**Figure 2:**
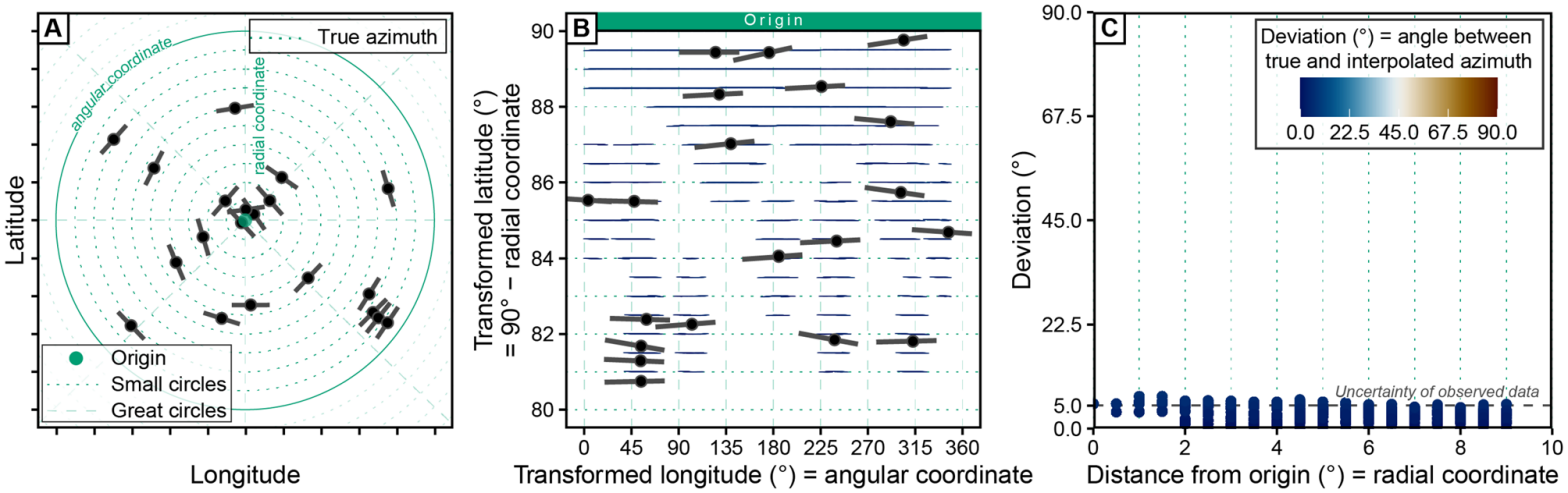
Stress-field predictions for a concentric stress field in a transformed coordinate reference system. (**A**) Identical dataset as in Fig. 1A but with the trajectories of the true azimuth of $\sigma _\text {Hmax}$ shown as small circles. The 1$^{\circ }$ small circles are centered at the origin of the stress source. (**B**) Stress interpolation of the same data in a transformed coordinate reference system (Mercator projection) by means of a general oblique transformation (i.e. stress origin is rotated into the North Pole of the map). Coordinates are related to the angular distance to the stress origin (i.e. the small circle or radial coordinate) and the angle measured along the small circle (angular coordinate). In this way, the small and great circles become straight lines and represent transformed latitudes and longitudes, respectively. The transformed orientation is expressed as the azimuthal deviation from the stress origin. The interpolation parameters are identical to Fig. 1. (**C**) The maximum deviation of the interpolated stress field compared with the uncertainty of the observed data.

Due to the small sample size, the highly variable $\sigma _\text {Hmax}$ orientations characterize a non-uniform stress field. The alignment of the $\sigma _\text {Hmax}$ orientation with the concentric rings around a center point, however, indicates that the orientation depends on the distance to the center point of the concentric geometry (Fig. 2A). Spatial interpolation averages the orientation between data points and thereby ignores the underlying geometrical feature of the stress field. Depending on the amount of averaged data, the averaged orientations will systematically deviate from the true orientation (red lines in Fig. 1B). Moreover, the systematic misfit, as shown by the large value of the azimuth deviation ($\gg 5^{\circ }$ ), increases towards the center of the point set where the orientation variability per area is the largest (Fig. 1C). This misfit might be avoided if a high number of data replicates the exact geometry of the underlying stress field. However, in many regions the geometry is unknown and stress or strain data are limited.

From the perspective of the center point, the $\sigma _\text {Hmax}$ data have similar orientations. Both the azimuth and its 5$^{\circ }$ scatter are independent of the distance to the center point (Fig. 2A). In contrast to the geographical CRS, this perspective describes a uniform stress field. This perspective is expressed geometrically by a coordinate transformation based on the radial distance to the center point and the azimuthal angle. The orientations are expressed by the azimuth deviation from the center point (Fig. 2B,C). In other words, the center point is used as the coordinate reference frame instead of the North Pole. Because the link between the geometrical feature of the stress field becomes the property of the new CRS, circular statistics do not suffer from the angle distortions of the stress-field geometry. Thereby, the deviation of the interpolated orientation does not exceed the 5$^{\circ }$ scatter of the input data indicating the good fit of the interpolation (Fig. 2C) and the averaged stress field in the transformed CRS outlines the expected uniform stress field, i.e., a constant orientation of $\sigma _\text {Hmax}$ (Fig. 2B). This simple test demonstrates how the geographical CRS (North Pole) can distort the actual geometry of the stresses. Hence, the geographical CRS does not allow for evaluating the location and orientation of the stresses with respect to their source (here the center point). If the stress source is known, a coordinate transformation into the perspective of this source provides a better and sample-size independent representation of the stress field. Such coordinate transformations, e.g. the oblique Mercator projection, are simple graphical approximations, that have been widely used to reconstruct plate motion (e.g. Refs.^22–28^) and, less commonly, to analyze stress fields^29,30^.

### The empirical link between plate motion and first-order stress

Most stress fields do not form concentric geometries and the stress originates from a more complex assemblage of forces and sources. Composite stress fields, however, can be decomposed into different orders of stresses depending on the wavelength of the field patterns^3,6,31,32^. The first-order component of a stress field represents $\ge$500 km wavelengths and second-order stress fields are characterized by shorter wavelengths between 100 and 500 km. Since each order is mainly controlled by specific forces^3,32^, the decomposition of a stress field allows for identifying the individual contribution of the stress sources. It has been shown that the first-order orientation of $\sigma _\text {Hmax}$ is predominantly subparallel to the relative motion of lithospheric plates^1,3,11,16,31–40^. This empirical correlation suggests that the first-order stress field is the result of the forces that drive and resist the motion of plates, i.e. plate boundary forces such as slab pull, trench suction, ridge push, collisional forces, and traction at the base of the lithosphere^41–44^.

This correlation is supported by the observed alignment of both the geodetic displacements (the elastic strain during the interseismic phase), and earthquake slip vectors (the permanent strain during the coseismic phase) with the direction of relative plate motion along plate boundary zones^22,45–49^. This geometrical link between strain and plate motion forms the foundation for the reconstruction of current^50–54^ and ancient plate motion^55,56^. Moreover, it provides a reference frame to analyze the stress field from the perspective of its first-order stress source, the plate boundary forces^16^.

### Combining spherical geometry and the first-order stress source

Any motion on a spherical surface is described as a rotation around an axis (Euler axis) intersecting the center of the sphere. The pole of rotation (PoR) or Euler pole denotes the location where the axis intersects the sphere. Both the magnitude and the direction of a force depend on the rotation which produces a torque. The net torque of two interacting plates, e.g. collisional or transform traction, is the sum of the torques of the two converging plates due to the conservation of the angular momentum of these plates^57^. The rotational axis of this resulting torque coincides with the Euler axis of relative plate motion^43^. Thus, horizontal resisting forces and their associated tectonic stresses oppose the relative motion on both sides of the plate boundary and act perpendicular and parallel to the strike of most of the convergent and transform plate boundaries, respectively^42,43^.

**Figure 3:**
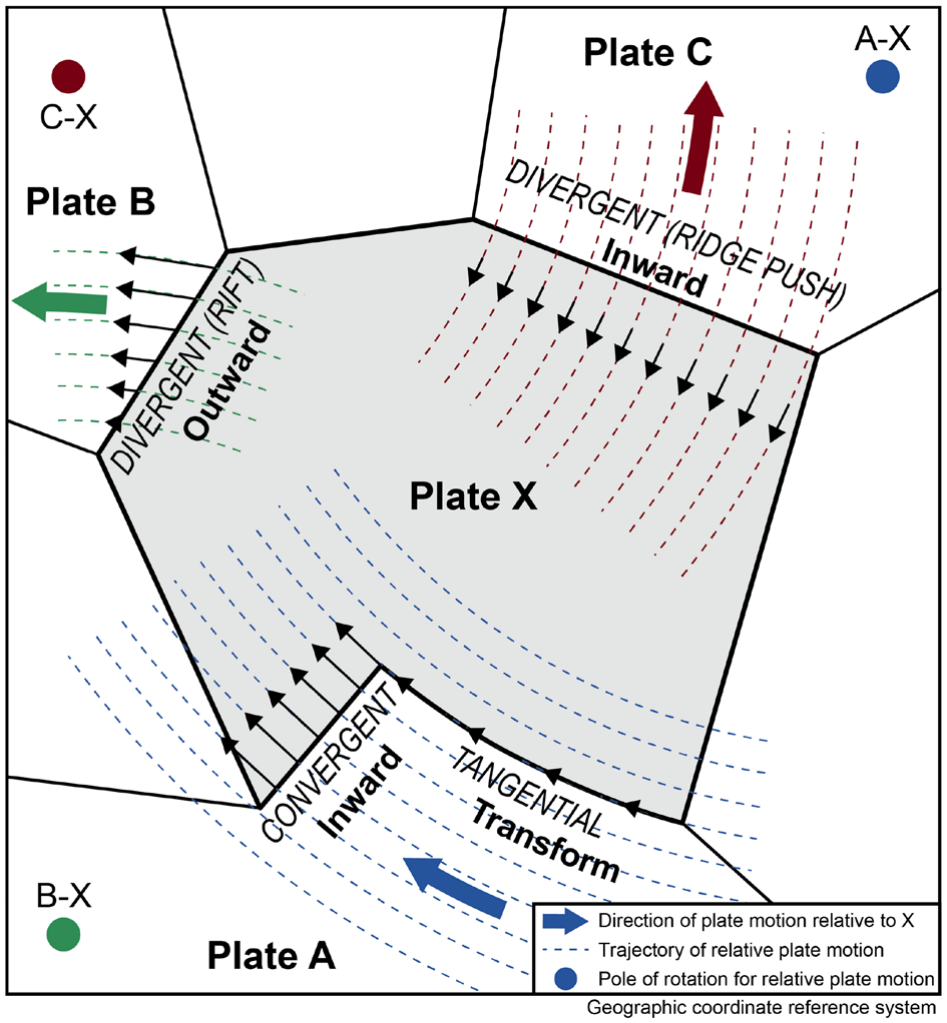
Sketch of the three types of displacement across plate boundaries due to relative plate motion. With respect to the interior of plate X, displacements across the boundaries of X are inward, outward, and tangentially in the direction of the motion of X relative to the neighboring plates (modified after Ref.^16^). Note that each boundary segment of X has differently oriented displacement trajectories (black arrows) and can have a different type of displacement. Outward displacement only occurs on the rift axis of a divergent plate boundary, but with increasing distance to the plate boundary, ridge push dominates. This creates an inward-moving plate boundary displacement.

**Figure 4:**
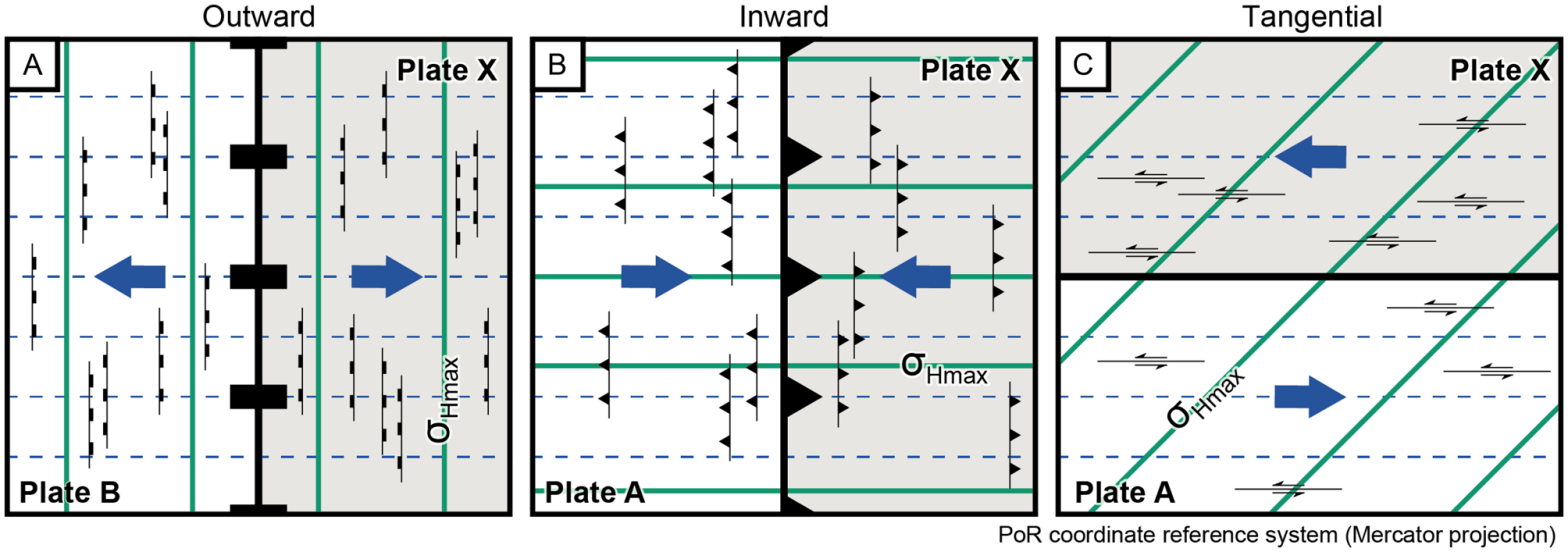
Sketch of the angular relationship between the direction of relative plate motion (blue arrows), the strike of faults (black lines), and the orientation of the maximum horizontal stress ($\sigma _\text {Hmax}$, colored solid lines) in the deforming area adjacent to the three types of displacement across plate boundaries: (**A**) $\sigma _\text {Hmax}$ is perpendicular to the direction of relative plate motion adjacent to an outward-moving boundary between two plates C and X. Predominant normal faults strike perpendicular to relative plate motion. Sketch shows the example of a divergent plate boundary (outward displacement only occurs on the rift axis of a divergent plate boundary. With increasing distance to the plate boundary, ridge push dominates. This creates an inward-directed plate boundary displacement where $\sigma _\text {Hmax}$ opposes the direction of relative plate motion. (**B**) $\sigma _\text {Hmax}$ is parallel to the direction of the relative plate motion adjacent to an inward-moving boundary between two plates A and X. Predominant thrust faults strike perpendicular to relative plate motion. (**C**) $\sigma _\text {Hmax}$ is at an angle of ±45$^{\circ }$ to the direction of the relative plate motion adjacent to the tangentially displaced boundary between two plates A and X. Predominant strike-slip faults strike parallel to relative plate motion. The trajectories of the relative plate motions (small circles) are displayed as stippled blue lines. Maps are shown in the Mercator projection of the PoR coordinate reference system.

**Figure 5:**
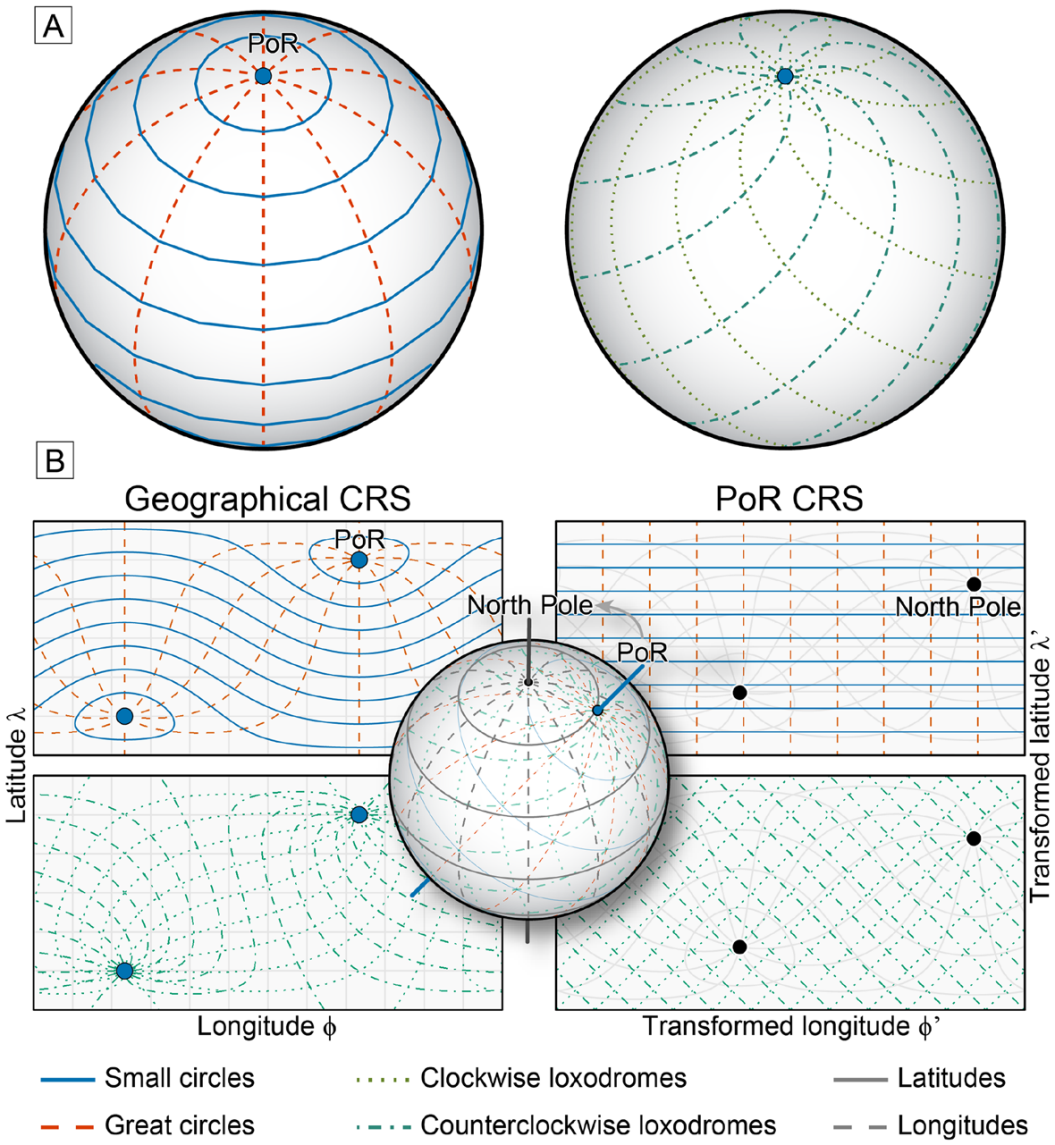
Geometries of stress trajectories. (**A**) Stress trajectories in an orthographic projection are viewed from an oblique angle to the pole of rotation, PoR (modified after Ref.^16^). Great circles are lines along the shortest distance between two data points. Small circles connect points with a constant distance to a point (e.g. PoR), producing concentric lines around that point. Loxodromes are lines of constant bearing that cut both small and great circles at a constant angle. (**B**) Exemplified geometries of stress trajectories. Left: conformal Mercator projections in the geographical coordinate reference system (CRS) (North Pole at the top of the map). Right: oblique Mercator projection in the PoR CRS with the PoR rotated to the top of the map. Inset visualizes the transformation between the two CRSs in orthographic projection.

There is an angular relationship between the plate motion direction and the orientation of the horizontal stress, which depends on the direction of displacement along the plate boundary^16^. The displacement is either directed outward or inward from the plate boundary, or tangentially along the boundary (Fig. 3). Respectively, $\sigma _\text {Hmax}$ is either perpendicular (Fig. 4A), parallel (Fig. 4B), or at an angle of $\pm 45^{\circ }$ to the relative motion of plates adjacent to the plate boundary (Fig. 4C). Thus, the trajectories of horizontal stress form three types of lines, which depend solely on the type of the plate boundary^16^. Considering the spherical geometry of Earth, these three geometries must follow spherical arcs on the Earth’s surface. These arcs describe great circles (lines along the shortest distance between the data point and the PoR of the relative plate motion), small circles (concentric lines around the PoR), and loxodromes (lines of constant bearing that cut both small and great circles at a constant angle) associated with inward-moving, outward-moving, and tangential displaced plate boundaries, respectively (Fig. 5).

Outward-moving plate boundaries produce tensional traction and displacements directed away from the plate interior (Fig. 3). For instance, on the mid-oceanic ridge, the stress field is predominantly controlled by the vertical push from the upwelling material and a horizontal pull that resists the spreading^45,58^. The resulting extension opposes the relative motion of the neighboring plates. Thus, the minimum horizontal stress ($\sigma _\text {hmin}$) is parallel to the divergence direction (Fig. 4A). Along spreading ridges and intracontinental rifting stresses are therefore dominated by normal faulting with $\sigma _\text {Hmax}$ trending perpendicular to the plate motion trajectories, i.e. along great circles passing through the data points and the PoR (Fig. 5).

Inward-moving plate boundaries induce compressional horizontal traction from the plate boundary towards the plate’s interior along the direction of relative plate motion (Fig. 3). Stresses across inward-moving plate boundaries are characterized by the dominance of thrusting or strike-slip faulting with $\sigma _\text {Hmax}$ trending parallel to plate convergence (Fig. 4B), i.e. parallel to small circles around the PoR of the relative plate motion (Fig. 5). Those stresses can be generated by convergent and divergent plate boundaries. Along convergent boundaries horizontal compression results from forces related to subduction and collision. With distance to divergent boundaries, in particular, mid-oceanic ridges, the increasing excess of the gravitational potential energy resulting from the elevated ridge creates horizontal compression, i.e. the ridge push.

Along tangentially displaced boundaries (transform boundaries), the two neighboring plates exert shear traction tangentially to the orientation of the boundary^42^. Faulting and displacement adjacent to these plate boundaries are characterized by strike-slip parallel to the plate motion, and thus, the principal axes of maximum and minimum stress are oriented at an angle of c. 45$^{\circ }$ and 135$^{\circ }$, respectively, to the plate motion (Fig. 4C). Geometrically, the $\sigma _\text {Hmax}$ orientation follows along 45$^{\circ }$ loxodromes which diverge—depending on the sense of the transform boundary—clockwise or counterclockwise from the PoR and intersect both small and great circles at a constant angle of 45$^{\circ }$ (Fig. 5).

## Methodology

Based on the outlined genetic and geometrical concept^16^, we developed a stress-field analysis model that comprises four consecutive steps: (i) extraction of the relative plate motion parameters that are linked to the tested plate boundary, (ii) transformation of the data point(s) into the PoR CRS, (iii) prediction of the direction of $\sigma _\text {Hmax}$ at the data point(s), and (iv) evaluation of the fit between the predicted direction and the observed direction of $\sigma _\text {Hmax}$, and its spatial correlation to the plate boundary. The mathematical operations of these four steps are briefly described in the following. The algorithms are implemented in the free and open-source software package tectonicr (see Supplementary Information online for details).

### Extraction of relative plate motion parameters

The theory for analyzing stress with respect to plate motion requires knowledge of the coordinates of the PoR associated with the relative motion between two plates. The transformation of the motion of a plate into its relative motion to another plate is done by the consecutive operation of rotations^59^.

Any point *P* on the Earth’s surface can be described as a vector $\vec {p}$ either given by its geographical latitude $\lambda$ and longitude $\phi$, or by its Cartesian coordinates *x*, *y*, and *z* (conversion between geographical and Cartesian coordinates is described in detail in the Supplementary Information online). A rotation on a sphere is defined by a vector that passes through the center of the sphere $\vec {e}$ where the vector intersects with the surface of the sphere (e.g. PoR), and a rotation angle $\omega$. Both parameters define the rotation $\text {Rot}(\omega , \vec {e})$. This 3D rotation is expressed in terms of quaternions^60^ as

1 $$\begin{aligned} q = \text {Sc}(q) + \text {Vec}(q) = \cos { \frac{\omega }{2}} + \vec {e} \, \sin {\frac{\omega }{2}} \end{aligned}$$

with $\text {Sc}(q)$ and $\text {Vec}(q)$ denoting the scalar and vector part of the quaternion *q* of unit norm. The inverse rotation of *q* is given by the conjugation defined as $q^* = \text {Sc}(q) - \text {Vec}(q) = \text {Rot}(-\omega , \vec {e})$. The rotation of a point $\vec {p}$ around $\vec {e}$ by $\omega$ to the point $\vec {p'}$ is

2 $$\begin{aligned} \text {Rot}(\omega , \vec {e}) \, \vec {p} = q\vec {p}q^{*} = \vec {p'}. \end{aligned}$$

A sequence of two consecutive Euler rotations $\text {Rot}(\omega _1, \vec {e}_1)$ followed by $\text {Rot}(\omega _2, \vec {e}_2)$ associated with $q_1$ and $q_2$ can be expressed as a single rotation $\text {Rot}(\omega , \vec {e}) = q_2 q_1$. Concatenation, however, is not commutative ($q_2 q_1 \ne q_1 q_2$) and a different order of consecutive rotations results in a different rotation.

The relative motion of plate B with respect to plate A is expressed by ${}_{A}\text {Rot}_{B}$. If the rotations ${}_{A}\text {Rot}_{B}$ and ${}_{A}\text {Rot}_{C}$ for plate C relative to A are known, the rotation ${}_{B}q_{C}$ for the C relative to B can be extracted from the two known rotations. In terms of quaternions, the rotation ${}_{B}q_{C}$ is provided by the concatenation of the quaternion ${}_{A}q_{C}$ with the conjugation of the quaternion ${}_{A}q_{B}$ associated with the two rotations, respectively:

3 $$\begin{aligned} {}_{B}q_{C} = {}_{A}q_{C} \; {{}_{A}q_{B}}^{*}. \end{aligned}$$

The rotation angle $\omega$ and the axis $\vec {e}$ of the resulting rotation are

4 $$\begin{aligned} \omega = 2 \arccos \bigl (\text {Sc}({}_{B}q_{C})\bigr ){} && \vec {e} = \frac{\text {Vec}({}_{B}q_{C})}{\sin {\frac{\omega }{2}}} \end{aligned}$$

This calculation allows for deriving the relative plate motions between neighboring plates. The coordinates of the PoR are given by the vector $\vec {e}$.

### Transformation of data points into the PoR coordinate reference system

The model^16^ predicts that the orientation of the first-order horizontal stress is aligned with horizontal trajectories of a plate boundary force. This implies that both the location and orientation (azimuth) of the stress data points can be described as seen from the perspective of the PoR. In other words, the PoR is used as a coordinate reference frame (Fig. 5). For the prediction of the $\sigma _\text {Hmax}$ orientation for a data point *P* and the calculation of its distance to the plate boundary, a coordinate transformation from the geographical CRS into the PoR CRS is required.

#### Coordinate transformation

For the conversion of the data location, we apply a general oblique transformation of the geographical CRS to describe the location of stress data points in PoR coordinates^22^. For the rotation of the coordinate reference frame, the coordinates of the PoR ($\lambda _\text {PoR}$, $\phi _\text {PoR}$) are used as translation parameters to rotate Earth in a way that the PoR will represent the map’s “North Pole” in the rotated projection (Fig. 5). Horizontal lines parallel the relative plate motion, and vertical lines are lines of great circles emanating from the rotation pole.

The transformation uses two rotations, $\text {Rot}(\omega _y, \vec {y})$ and $\text {Rot}(\omega _z, \vec {z})$, around the y-axis $\vec {y} = (0, 1, 0)$ and z-axis $\vec {z} = (0, 0, 1)$ by the angles $\omega _y = 90^{\circ }-\lambda _\text {PoR}$ and $\omega _z = 180^{\circ }-\phi _\text {PoR}$, respectively. The rotations $\text {Rot}(\omega _y, \vec {y})$ and $\text {Rot}(\omega _z, \vec {z})$ are given in terms of quaternions $q_y$ and $q_z$, respectively, by

5 $$\begin{aligned} q_y&= \cos \left( \frac{\omega _y}{2}\right) + \sin \left( \frac{\omega _y}{2}\right) \vec {y} \end{aligned}$$

6 $$\begin{aligned} q_z&= \cos \left( \frac{\omega _z}{2}\right) + \sin \left( \frac{\omega _z}{2}\right) \vec {z} \end{aligned}$$

The transformation of a data point *P* from the geographical CRS (expressed as vector $\vec {p}$) to $P'$ into the PoR CRS ($\vec {p'}$) is described by

7 $$\begin{aligned} \vec {p'} = q_y q_z \; \vec {p} \; (q_y q_z)^{*} \end{aligned}$$

The transformation of the data point from the PoR into the geographical CRS is

8 $$\begin{aligned} \vec {p} = (q_y q_z)^{*} \; \vec {p'} \; q_y q_z \end{aligned}$$

#### Azimuth transformation

**Figure 6:**
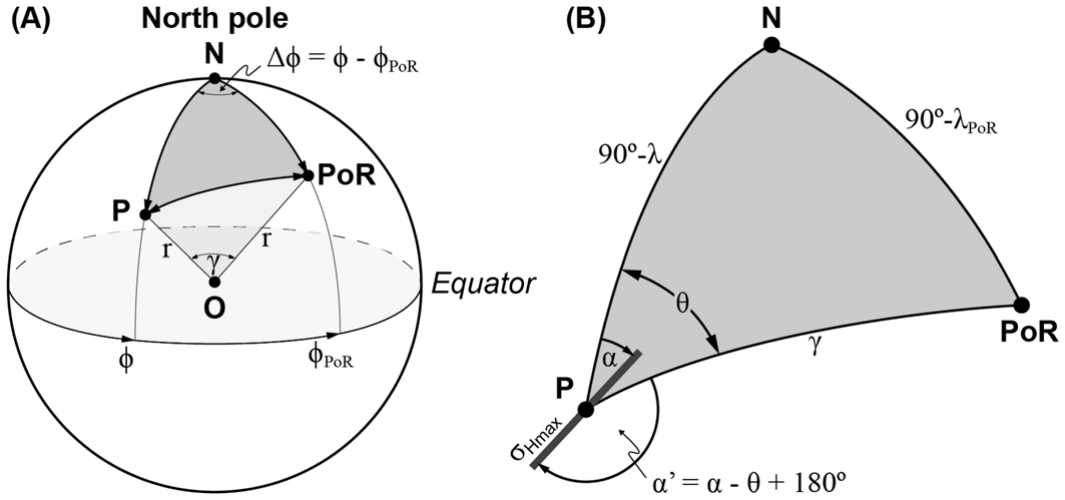
(**A**) Geometry for the determination of the angular distance along the great circle between point P and the pole of rotation PoR (*N* North Pole, *O* center of the Earth, *r* Earth’s radius, modified after Ref.^61^). (**B**) Angular relations of the spherical triangle in (A) which are used in deriving the transformed $\sigma _\text {Hmax}$ azimuth $\alpha '$ (modified after Ref.^46^).

The North Pole, the PoR, and the data point *P* define a spherical triangle with sides (great circles) $90^\circ -\lambda$, $90^\circ -\lambda _\text {PoR}$, and $\gamma$, where $\lambda _\text {PoR}$ is the latitude of the PoR (Fig. 6A). The angle $\gamma$ is the great circle distance between *P* and the PoR and is equivalent to the transformed latitude of *P* in the PoR CRS (Fig. 5), i.e. $\gamma = |90^\circ - \lambda ' |$. Using the law of cosines from spherical trigonometry^46^, the angle $\gamma$ is derived from

9 $$\begin{aligned} \cos \gamma= \cos (90^\circ - \lambda _\text {PoR}) \; \cos (90^\circ - \lambda ) + \sin (90^\circ - \lambda _\text {PoR}) \; \sin (90^\circ - \lambda ) \; \cos {\Delta \phi } \end{aligned}$$

10 $$\begin{aligned}= \sin {\lambda _\text {PoR}} \; \sin {\lambda } + \cos {\lambda _\text {PoR}} \; \cos {\lambda } \; \cos {\Delta \phi } \end{aligned}$$

where $\Delta \phi$ is the angle between the meridians passing through *P* and the PoR, i.e. the longitudinal difference of *P* and PoR. The spherical triangle contains the angle $\theta$, that is the angle measured between the two great circles from *P* to the geographic North Pole and the PoR (Fig. 6B). Using the spherical law of sines, the angle $\theta$ is derived from

11 $$\begin{aligned} \sin \theta = \frac{\sin {\Delta \phi } \; \sin {(90^\circ - \lambda _\text {PoR})}}{\sin {\gamma }} = \frac{\sin {\Delta \phi } \; \cos {\lambda _\text {PoR}}}{\sin {\gamma }} \end{aligned}$$

The angle $\theta$ describes the direction of the PoR from point *P* and can be used for the conversion of the $\sigma _\text {Hmax}$ azimuth into the PoR CRS. The transformed azimuth $\alpha '$ at *P* is the angular difference between the azimuth $\alpha$ at *P* in the geographical CRS and the great circle that passes through *P* and the PoR:

12 $$\begin{aligned} \alpha ' = \alpha - \theta + 180^\circ \end{aligned}$$

The quantities used in these formulas (Eqs. 9, 10, 11 and 12) are shown in Fig. 6.

### Predicted direction of horizontal stress

In the PoR CRS, the predicted azimuth $\beta '$ of $\sigma _\text {Hmax}$ is either 0$^{\circ }$, 90$^{\circ }$ or $\pm 45^{\circ }$ for all possible locations. The angle is linked to the displacement type of the tested plate boundary, i.e. outward, inward, or tangentially displaced plate boundary, respectively (Table 1). The predicted azimuth can now be compared with the transformed azimuth $\alpha '$ of $\sigma _\text {Hmax}$. In both coordinate reference systems, the deviation of the observed orientation $\alpha$ (or $\alpha '$) from the predicted orientation $\beta$ (or $\beta '$) of $\sigma _\text {Hmax}$ at *P* is identical and expressed as follows:

13 $$\begin{aligned} \Delta \alpha = \alpha - \beta{} && \Delta \alpha ' = \alpha ' - \beta ' \end{aligned}$$

Because $\Delta \alpha = \Delta \alpha '$, the great circle orientation $\theta$ is also identical in both the geographical CRS and the PoR CRS, i.e. $\theta = \theta '$. Therefore, the predicted azimuth $\beta$ at point *P* in the geographical CRS is given by:

14 $$\begin{aligned} \beta = \theta + \beta ' - 180^\circ \end{aligned}$$

**Table 1:** Predicted azimuth ($\beta$) of maximum horizontal stress ($\sigma _\text {Hmax}$) adjacent to the various plate boundary types in the geographical coordinate reference system.

Displacement of plate boundary	Stress regime	$\sigma _\text {Hmax}$ azimuth	Geometry of trajectories
Outward	Normal fault	$\beta = \theta$	Great circles
Tangential (L)	Strike-slip (L)	$\beta = \theta + 45^{\circ }$	Counterclockwise loxodromes
Inward	Thrust	$\beta = \theta + 90^{\circ }$	Small circles
Tangential (R)	Strike-slip (R)	$\beta = \theta + 135^{\circ }$	Clockwise loxodromes

The minimum horizontal stress is perpendicular to $\beta$. Hence, it follows the trajectories perpendicular to those predicted for $\sigma _\text {Hmax}$.

*L* left-lateral,* R* right-lateral.

### Evaluation of the fit between the predicted and observed data

#### Statistical measure for the fit

To evaluate the fit between the predicted and the observed direction of $\sigma _\text {Hmax}$, we use the normalized $\chi ^2$ criterion^16^. This statistical test quantitatively evaluates the significance of the predicted $\sigma _\text {Hmax}$ for the observed stress direction relative to their reported standard deviation:

15 $$\begin{aligned} \text {Norm}~\chi ^2 = \frac{\sum _{i=1}^{n} \left( \frac{ |\Delta \alpha _i |}{\sigma _i}\right) ^2}{\sum _{i=1}^{n} \left( \frac{90^{\circ }}{\sigma _i}\right) ^2} \end{aligned}$$

with the $$\sigma _i$$ parameter being the reported uncertainty of the observed azimuth and *n* representing the number of observations that are used in each test. The normalized $$\chi ^2$$ test yields a number between 0 and 1, which represent the quality of the fit. Low values ($$\le 0.15$$ indicate good agreement between predicted and observed directions. Large values ($$>0.7$$) indicate a systematic misfit between predicted and observed directions of about 90$$^{\circ }$$. Random distribution of $$\sigma _\text {Hmax}$$ directions results in $$\text {Norm}~\chi ^2$$$$= 0.33$$. An example of this goodness-of-fit evaluation can be seen for the concentric stress field in the geographic and the PoR CRS (Figs. 1C and 2C). Alternative statistical estimators for circular dispersion and goodness-of-fit tests, such as the Rayleigh test and Watson’s U^2^ test^62, 63^, can be used after tranforming the azimuths into the PoR CRS.

#### Variation of the fit with distance to the plate boundary

In the last step of our stress-analysis theory we evaluate the stress data with respect to their distance to the associated plate boundary. The transformation of stress data into the PoR CRS allows extracting the distance of a data point from the tested plate boundary measured along these trajectories. In the PoR CRS, the distance is simply the coordinate difference between the great or small circles that separate the data point from the inward/outward or tangentially displaced boundary, respectively (Fig. 2A).

Two steps are required to measure the distance. First, the coordinates of the data point ($\lambda , \phi$) and the plate boundary ($\lambda _\text {pb}, \phi _\text {pb}$) are transformed into the PoR CRS. Next, the distance is calculated using the coordinate difference between the transformed point ($\lambda ', \phi '$) and the transformed plate boundary ($\lambda '_\text {pb}, \phi '_\text {pb}$). In this way, the angular distance is given by the longitudinal difference between the plate boundary and the data point for inward and outward-moving plate boundaries $\Delta \phi ' = \phi '_\text {pb} - \phi '$. For tangentially displaced plate boundaries, the angular distance is given by their latitudinal difference $\Delta \lambda ' = \lambda '_\text {pb} - \lambda '$. The longitudinal and latitudinal differences in the PoR CRS represent small circle and great circle distances. The great circle distance for expressing the distance to a tangential plate boundary is the product of $\Delta \lambda '$ and the Earth’s radius *r*:

16 $$\begin{aligned} s_\text {tan} = \Delta \lambda ' \, r. \end{aligned}$$

The small circle distance for inward and outward-moving plate boundaries is

17 $$\begin{aligned} s_\text {in/out} = \Delta \phi ' \, r \, \cos {\lambda '}. \end{aligned}$$

Taking together the proposed evaluation of the fit between the predicted and the observed stress orientations and their spatial relationship with respect to the plate boundary allows for estimating the width of the plate boundary zone (*D*). Thereby, *D* is obtained by the maximum distance *s* between the plate boundary and the data points where $\text {Norm}~\chi ^2$ is smaller than a given threshold (e.g. 0.15).

## Testing the theory

The theory for stress-field analysis and the capabilities of the software tectonicr are demonstrated using the stress fields of the San Andreas Faul–Gulf of California area, Central Asia, the North Atlantic Ridge–Iceland area, and the global stress field. For these applications, we use the A, B, C, and D quality-ranked data of the WSM2016 dataset^17^. Measurement uncertainties are represented by the reported uncertainties and, if that information is not available, the uncertainty (1$\sigma$ standard deviation) that is associated with the quality of the data (A is ±15$^{\circ }$, B is ±20$^{\circ }$, C ±25$^{\circ }$, D ±40$^{\circ }$). The geometries used for plate boundaries are based on Bird^64^.

The parameters for the current plate motion are extracted from the models NUVEL-1A^49,51^, NNR-MORVEL56^27,52^, REVEL^53^, and GSRM v2.1^54^. All motion parameters are transferred into relative plate motion parameters of neighboring plates, i.e. the Pacific and North America for the area of San Andreas Faul–Gulf of California, India and Eurasia for Central Asia, and North America and Eurasia for the area of the North Atlantic Ridge and Iceland, respectively. The direction of North America’s and Eurasia’s absolute plate motion (hotspot reference frame HS3-NUVEL-1A^65^) is additionally compared with the stress orientation of the San Andreas Faul–Gulf of California area and Central Asia, respectively, to estimate the contribution of basal drag on the stress state of the deforming area. The PoR coordinates and the statistical results of the tests are shown in Table 2. The used and generated datasets can be found in the Supplementary Material online. The tests can be reproduced with the R package tectonicr as described in detail in the Supplementary Information online.

**Table 2:** Statistical parameters and test results for different recent plate motion models.

Model	Pole of rotation			Azimuth^a^ ($^{\circ }$)		Norm	Distance to	Max.
	Lat. ($^{\circ }$)	Lon. ($^{\circ }$)	Rate ($^{\circ }$/Myr)	$\alpha '$^b^	$\beta '$^c^	$\chi ^2$	PoR ($^{\circ }$)	$\sigma (\beta )$^d^ ($^{\circ }$)
*San Andreas Fault – Gulf of California (N*^e^*: 1082)*								
NUVEL-1A	– 48.7	101.8	0.75	$137.0 \pm 12.7$	135	0.033	51 – 67	0.8
MORVEL56	– 48.9	108.2	0.75	$135.6 \pm 12.9$	135	0.033	47 – 63	1.0
GSRM 2.1	– 49.3	103.9	0.79	$137.1 \pm 12.9$	135	0.033	50 – 65	1.0
REVEL	– 50.4	107.8	0.75	$137.7 \pm 12.8$	135	0.035	47 – 63	2.7
HS3-NUVEL-1A	– 74.7	13.4	0.38	$31.9 \pm 18.5$	90	0.544	51 – 67	
*Himalaya – Tibet (N: 1047)*								
NUVEL-1A	24.6	18.0	0.51	$95.1 \pm 20.6$	90	0.107	19 – 42	4.3
MORVEL56	31.8	17.4	0.48	$88.0 \pm 21.0$	90	0.078	23 – 44	4.6
GSRM 2.1	27.3	17.6	0.40	$82.2 \pm 20.6$	90	0.096	20 – 43	3.7
REVEL	28.6	11.7	0.36	$88.7 \pm 21.3$	90	0.081	19 – 39	6.4
HS3-NUVEL-1A	– 61.9	73.5	0.20	$8.6 \pm 22.2$	90	0.468	19 – 41	
*North Atlantic Ridge – Iceland (N: 342)*								
NUVEL-1A	– 62.3	– 43.5	0.21	$2.9 \pm 29.0$	0	0.192	35 – 44	1.0
MORVEL56	– 61.7	– 40.5	0.21	$4.4 \pm 27.5$	0	0.189	34 – 43	2.5
GSRM 2.1	– 70.7	– 58.9	0.23	$-0.9 \pm 26.3$	0	0.202	44 – 54	1.3
REVEL	– 68.0	– 43.4	0.24	$4.9 \pm 27.3$	0	0.188	40 – 50	1.5

^a^Orientations of the maximum horizontal stress are given in the transformed PoR coordinate reference system.

^b^Observed azimuth given as quality-weighted circular median and interquartile range.

^c^Predicted azimuth.

^d^Maximum error of the prediction of the azimuth (Eq. 18).

^e^Number of data.

### Tangentially displaced plate boundary: San Andreas Fault–Gulf of California

**Figure 7:**
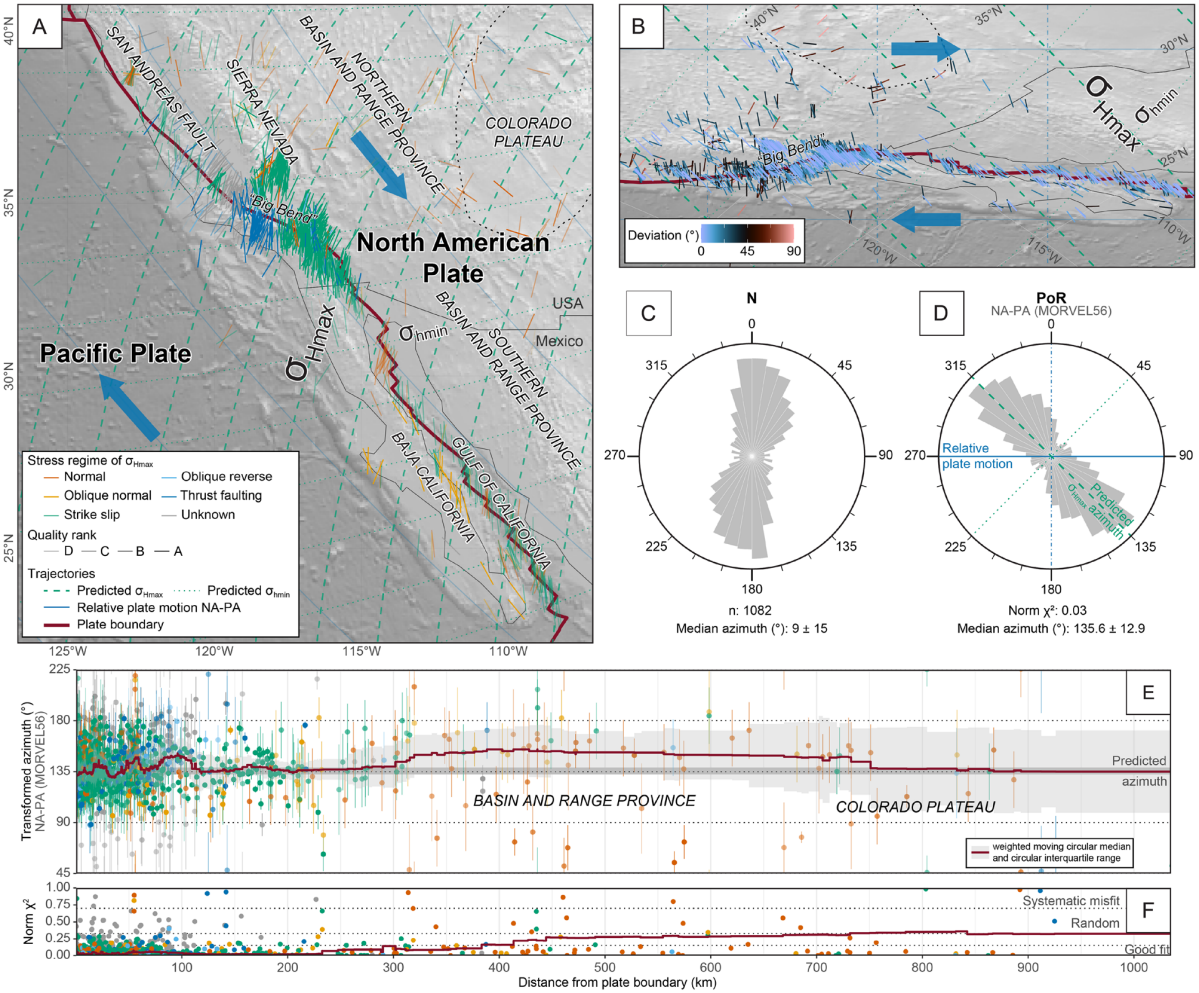
Stress field adjacent to the San Andreas Fault–Gulf of California segment of the plate boundary between the Pacific (PA) and the North America (NA) plates (based on NNR-MORVEL56). (**A**) Stress orientation and regime of the maximum horizontal stress ($\sigma _\text {Hmax}$) in the geographical coordinate reference system (Mercator projection). The shaded relief is based on ETOPO1^74^. (**B**) Orientation of the same $\sigma _\text {Hmax}$ shown in (**A**) transformed into the PoR coordinate reference system (Mercator projection). Color of the $\sigma _\text {Hmax}$ axes indicates the deviation of the observed $\sigma _\text {Hmax}$ azimuth from the predicted $\sigma _\text {Hmax}$ azimuth. (**C** and** D**) Equal-area rose diagrams showing the frequency distribution of the $\sigma _\text {Hmax}$ orientation in the geographical (**C**) and PoR coordinate reference system (**D**). Frequencies are weighted by the reported uncertainties and the optimal bin-width^75^ for both rose diagrams is 9$^{\circ }$. (**E**) Orientation of transformed $\sigma _\text {Hmax}$ and the range of the reported standard deviation (1$\sigma$) as a function of the distance to the plate boundary. Data are color coded according to the legend in (**A**). (**F**) Results of the $\text {Norm}~\chi ^2$ test as a function of the distance to the plate boundary.

The area of the San Andreas Fault and the Gulf of California comprises a large amount of available stress data (n = 1082) and regional geology is well constrained. The area is affected by the plate boundary between the plates of the Pacific and North America. The San Andreas Fault is a ca. 4000 km long segment of this plate boundary and constitutes a generally dextral strike-slip fault with $>100$ km of tangential displacement since Miocene times^66,67^. The plate boundary stress field is characterized by NNE-SSW compression (Fig. 7). The orientation of $\sigma _\text {Hmax}$ is fairly uniform over a 100–500 km lateral extent. This is interpreted to be indicative of plate boundary forces that provide the majority of the total stress field^68^. Adjacent to the Big Bend segment of the San-Andreas Fault in southern California (Fig. 7A) thrust faulting occurs with $\sigma _\text {Hmax}$ oriented nearly perpendicular to the strike of the San-Andreas Fault^30,68–70^. The stress field to the NW of the Sierra Nevada differs from areas adjacent to the plate boundary due to the dominant occurrence of extensional stresses with W–E to WNW–ESE trending $\sigma _\text {Hmax}$^1^. Here, the stresses are deflected along the margin of the tectonically stable Colorado Plateau^71^. The plateau separates the Basin and Range Province into a northern and southern part (Fig. 7). The dominant extensional stresses of the Basin and Range Province are interpreted to be generated by internal buoyancy forces due to lateral density gradients and topography^72,73^.

We test the deforming San Andreas Fault–Gulf of California area against the expected stress trajectories that are associated with the forces generated by dextral motion along the plate boundary (Fig. 3). We expect a good fit close to the plate boundary and a systematic misfit farther away, in particularly in the Basin and Range Province and the Colorado Plateau. Additionally, we test the same stress dataset against different publicly available models for current plate motion. Ultimately, we subtract the predicted first-order stress from the observed stress field to identify lower-order stress-field constituents. These resulting lower-order stress fields can be evaluated using known geological information from the area.

Because the transform plate boundary has a right-lateral tangential displacement, $\sigma _\text {Hmax}$ is expected to be oriented along counterclockwise loxodromes passing through the PoR associated with the relative plate motion (Figs. 4 and 5). Hence, the predicted orientation is 135$^{\circ }$ in the transformed CRS (Table 1). Our test reveals a good fit between the predicted and the observed $\sigma _\text {Hmax}$ orientation (Fig. 7). The average transformed azimuth is $135.6 \pm 12.9^{\circ }$ and the small $\text {Norm}~\chi ^2$ value of 0.03 confirms the good fit. Close to the plate boundary, there is a wide scatter of azimuths, but the average of the data is ca. 135$^{\circ }$ at distances of 0–300 km from the boundary. Therefore, the scatter may result from the high concentration of data near the boundary and their random scattering. Oblique stress regimes such as those associated with oblique normal faults in the Gulf area, only slightly deviate from the predicted orientation.

The small average azimuth variability of $\pm 12.9^{\circ }$ (circular interquartile range (IQR $=25.8^{\circ }$) in the transformed CRS indicates a uniform stress field. The average azimuth variability in the geographical CRS is larger (IQR $=33.8^{\circ }$). This indicates that the rotation from an N-S orientation towards a NE-SW orientation of $\sigma _\text {Hmax}$ does not reflect a change of stress sources (Fig. 7). The apparent rotation is rather a result of angle distortion in the geographical CRS since both orientations are aligned with the predicted trajectories.

Although there is a generally good fit for up to ca. 700 km from the boundary, the data becomes noisier with increasing distance. In particular, stresses in a normal fault regime deviate from the predicted orientation (Fig. 7E and F), suggesting that the dominant stress sources in the Colorado plateau are not related to the plate boundary between North America and the Pacific plate.

Based on $\text {Norm}~\chi ^2$ criteria, the predictions from all four relative plate motion models yield good fits with $\text {Norm}~\chi ^2$ $\le 0.034$ (Table 2). The direction of the absolute plate motion is not aligned with the $\sigma _\text {Hmax}$ orientations as indicated by the large deviation of the observed orientation ($32\pm 19^{\circ }$) from the predicted 90$^{\circ }$, and the large $\text {Norm}~\chi ^2$ value of 0.544. The transformation of the stress data and fault geometries into the PoR CRS (Fig. 7B) demonstrates that the Big Bend segment of the San Andreas Fault strikes slightly obliquely to the small circles of the relative plate motion. Because $\sigma _\text {Hmax}$ does not deviate from the loxodrome trajectories (45$^{\circ }$ to these small circles), the consequently increased fault-perpendicular compression results in transpression and a thrust-fault stress regime^30,68–70,76,77^.

**Figure 8:**
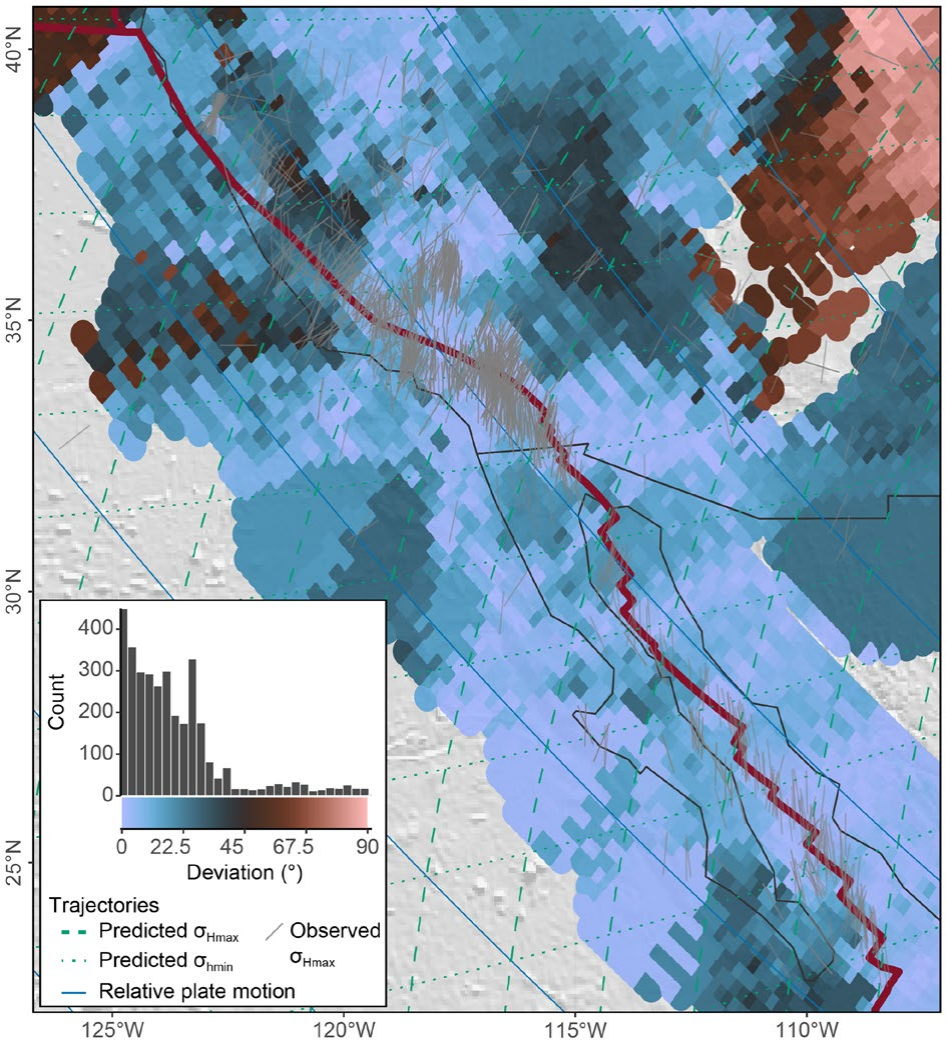
Deviation of the direction of the smoothed stress field from the predicted first-order stress field of the San Andreas Faul–Gulf of California area. Inset shows the distribution of deviation angles. Interpolation parameters: algorithm: stress2grid^21^; grid size: 0.25$^{\circ }$; search radius: 50 – 350 km; minimum amount of data in search radius: 3; weighting: inverse distance and quality. The shaded relief is based on ETOPO1^74^.

The last step of our stress analysis aims to identify regions where the assumed plate boundary forces do not control the $\sigma _\text {Hmax}$ orientation. Figure 8 shows the deviation of the azimuths of a large spatially-resolved (0.25$^{\circ }$ spacing) interpolated stress field from the first-order stress direction predicted by the model (the interpolated stress field is shown in Supplementary Fig. S1 online). In the example of the San Andreas Fault–Gulf of California area, almost the entire region including the Basin and Range Province is characterized by a correlation between the interpolated and the predicted stress orientation (deviation $<25^{\circ }$, blue colors in Fig. 8).

However, there is a stress anomaly that shows a substantial deviation ($>40^{\circ }$) between the predicted and observed stress directions, namely the Colorado Plateau (red colors in Fig. 8). Here, WNW-ESE trending $\sigma _\text {Hmax}$ deviates from the predicted $\sigma _\text {Hmax}$ orientation suggesting that plate boundary forces, i.e. transform traction, do not control the orientation of stress along the margin of the Colorado Plateau.

### Inward-moving plate boundary: Himalaya–Tibet

**Figure 9:**
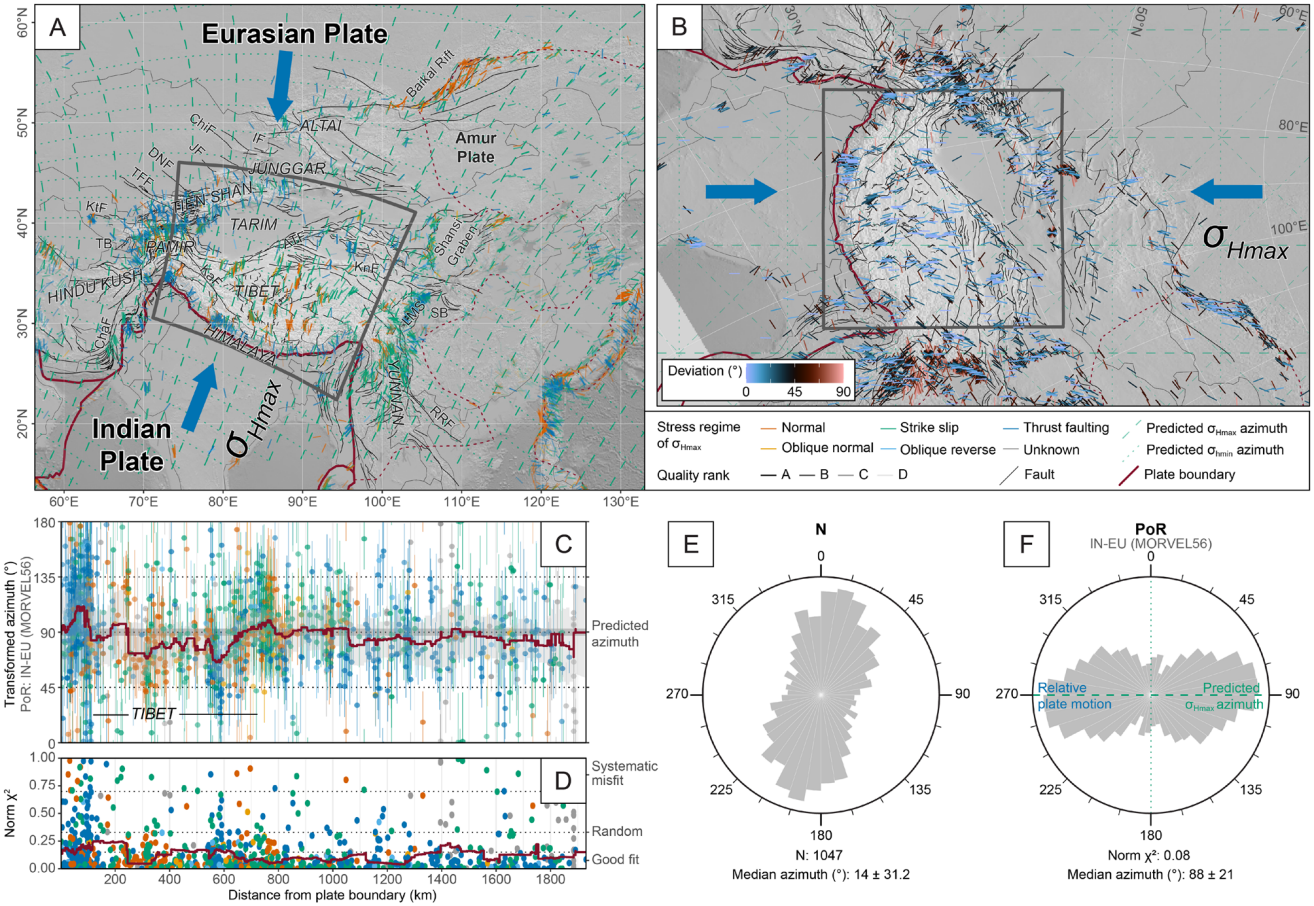
Stress field of Central Asia. Stress trajectories are modeled from the relative plate motion between the Indian (IN) and the Eurasian (EU) plates (based on NNR-MORVEL56). See Fig. 7 for details. Statistics (**C**–**F**) are based on the Himalaya–Tibet region (gray rectangle in (**A**) and (** B**)). Bin-width of rose diagrams: 9$^{\circ }$. Faults (adapted from Ref.^84^): *ATF* Altyn Tagh Fault, *ChaF* Chaman Fault, *ChiF* Chingiz Fault, *DNF* Dzhalair-Naiman Fault, *IF* Irtysh Fault, *JF* Junggar Fault, *KaF* Karakorum Fault, *KnF* Kunlun Fault, *KtF* Kuldzukhtau Fault, *LMS* Longmenshan Thrust Belt, *RRF* Red River Fault,*TFF* Talas-Fergana Fault. Basins: *SB* Sichuan Basin, *TB* Tadjik Basin.

The present-day deformation in Central Asia is driven by the indention of the Indian plate into the Eurasian plate^78–80^. Both plates currently converge at a rate of up to c. 60 mm/yr. The indention results in a frontal collision zone (Himalaya), and two dextral and sinistral transfer zones in Pakistan and Myanmar, respectively, that mark the plate boundary between the two plates (Fig. 9). The widespread deformation due to the collision extends up to the Baikal Sea region, i.e. $>3000$ km away from the plate boundary in the Himalaya^79^. The associated stress field is dominated by ca. N-S trending $\sigma _\text {Hmax}$ (median orientation of $14^{\circ } \pm 31^{\circ }$). In the Himalaya, the stress is characterized by a thrust-fault regime. Strike-slip and normal-fault stress regimes are predominant in the Tibetan Plateau and the associated $\sigma _\text {Hmax}$ directions are parallel to $\sigma _\text {Hmax}$ of thrusts in the Himalaya. The orientations of $\sigma _\text {Hmax}$ associated with strike-slip faulting along the western and eastern margins of the Indian plate, however, are ca. NW-SE and NE-SW, respectively. GPS velocities and geological constraints indicate that the Tibetan Plateau reaches west and particularly east of the north projected Indian plate margins due to the growth of the plateau (e.g. Refs.^81–83^). Those lateral crustal movements result in obliquely directed deformation (with respect to the convergence direction between India and Eurasia) in the Hindukush Range–Tadjik Basin and the Longmenshan Thrust Belt–Sichuan Basin, respectively (Fig. 9).

We test the stress field of the Himalaya and Tibet (grey box in Fig. 9A and B) against an inward-moving plate boundary (Fig. 3) because of the convergent character of the plate boundary between India and Eurasia. Therefore, $\sigma _\text {Hmax}$ is expected to be oriented parallel to the convergence direction (Fig. 4) that forms small circles around the PoR associated with the relative plate motion (Fig. 5). Hence, the predicted orientation is 90$^{\circ }$ in the transformed CRS (Table 1). We expect a good fit close to the convergent plate boundary and an increasing misfit farther away. Further misfits are expected to occur along the Hindukush Range–Tadjik Basin and Longmenshan Thrust Belt–Sichuan Basin, as well as along the strike-slip boundary between India and Eurasia in Pakistan and Myanmar.

The test reveals a statistically good fit for the Himalaya–Tibet area ($\text {Norm}~\chi ^2$: 0.08), which covers a range of ca. 2000 km from the plate boundary (Table 2). A generally acceptable fit is obtained for the broader area of Central Asia ($\text {Norm}~\chi ^2$: 0.26) reaching as far as ca. 3000 km from the plate boundary (up to the Baikal Rift zone, Fig. 9). Based on $\text {Norm}~\chi ^2$ criteria, the predictions from all four relative plate motion models yield good fits with $\text {Norm}~\chi ^2$ $\le 0.107$ (Table 2). The direction of absolute plate motion, however, seems not to be aligned with the $\sigma _\text {Hmax}$ orientations as indicated by the large deviation of the observed orientation ($32\pm 19$ $^{\circ }$) from the predicted orientation (90$^{\circ }$). The misfit is supported by the large $\text {Norm}~\chi ^2$ test value of 0.468.

The orientations of $\sigma _\text {Hmax}$ associated with thrusts and strike-slip faults are parallel to the predicted small circle geometries parallel to the convergence direction (Fig. 9). This implies that $\sigma _\text {Hmax}$ approaches the maximum principal stress axis. In the elevated area of the Tibetan Plateau, however, the $\sigma _\text {Hmax}$ direction related to normal faults is also parallel to the convergence direction. This supports that here the maximum principal stress axis is vertical due to the over-thickened crust leading to extension perpendicular to the convergence^85–87^. The coordinate transformation reveals the geometrical relationship of the Central Asian faults to the India-Eurasia collision (Fig. 9B). For instance, strike-slip faults of the Tien Shan (e.g. faults of Talas Fergana, Dzhalair-Naiman, and Junggar) and of the Altai comprise an en-echelon set of dextral strike-slip faults^78^ that strike parallel to counterclockwise loxodromes passing through the PoR. The sinistral Altyn Tagh Fault represents the conjugate fault to those dextral faults and, thus, follows clockwise loxodromes^78^. The extensional faults of the Baikal Rift and the Shansi Graben are subparallel to the convergence (small circle trajectories). The test reveals that stresses from the India-Eurasia collision are transferred far into the Eurasian lithosphere.

**Figure 10:**
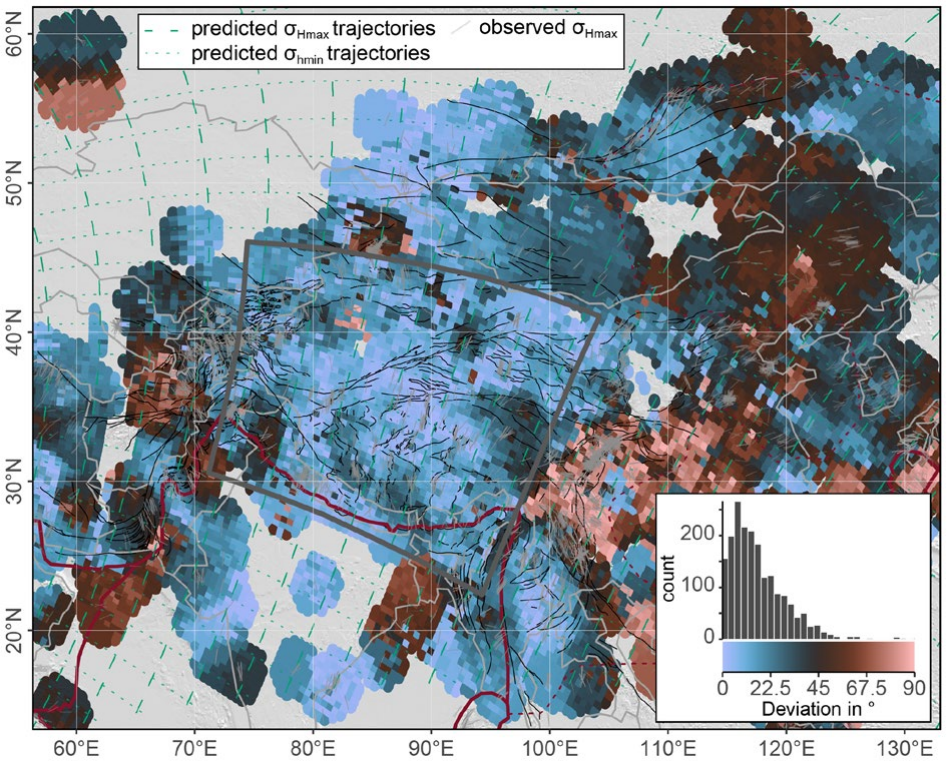
Deviation of the direction of the smoothed stress field from the predicted first-order stress field of Central Asia. Inset shows the distribution of the deviation angles of the Himalaya–Tibet area (gray rectangle). See Fig. 8 for interpolation parameters and map details.

The interpolation of the stress field in the PoR system highlights stress anomalies in Central Asia (Fig. 10, the interpolated stress field is shown in Supplementary Fig. S1 online). Deviations up to 45$^{\circ }$ occur along the eastern and western margins of the Indian plate where $\sigma _\text {Hmax}$ rather follows loxodromes around the PoR. Along the Longmenshan Thrust Belt–Sichuan Basin and Hindu Kush–Tadjik Basin, $\sigma _\text {Hmax}$ is perpendicular to the predicted orientation from the convergence supporting that the oblique stress directions are generated by the lateral escape motion due to the gravitational collapse of the elevated and overthickened Tibetan crust. Further large stress deviations (up to 90$^{\circ }$) exist in the rigid blocks of Tarim and Jungger^88^.

### Outward-moving plate boundary: North Atlantic Ridge and Iceland

**Figure 11:**
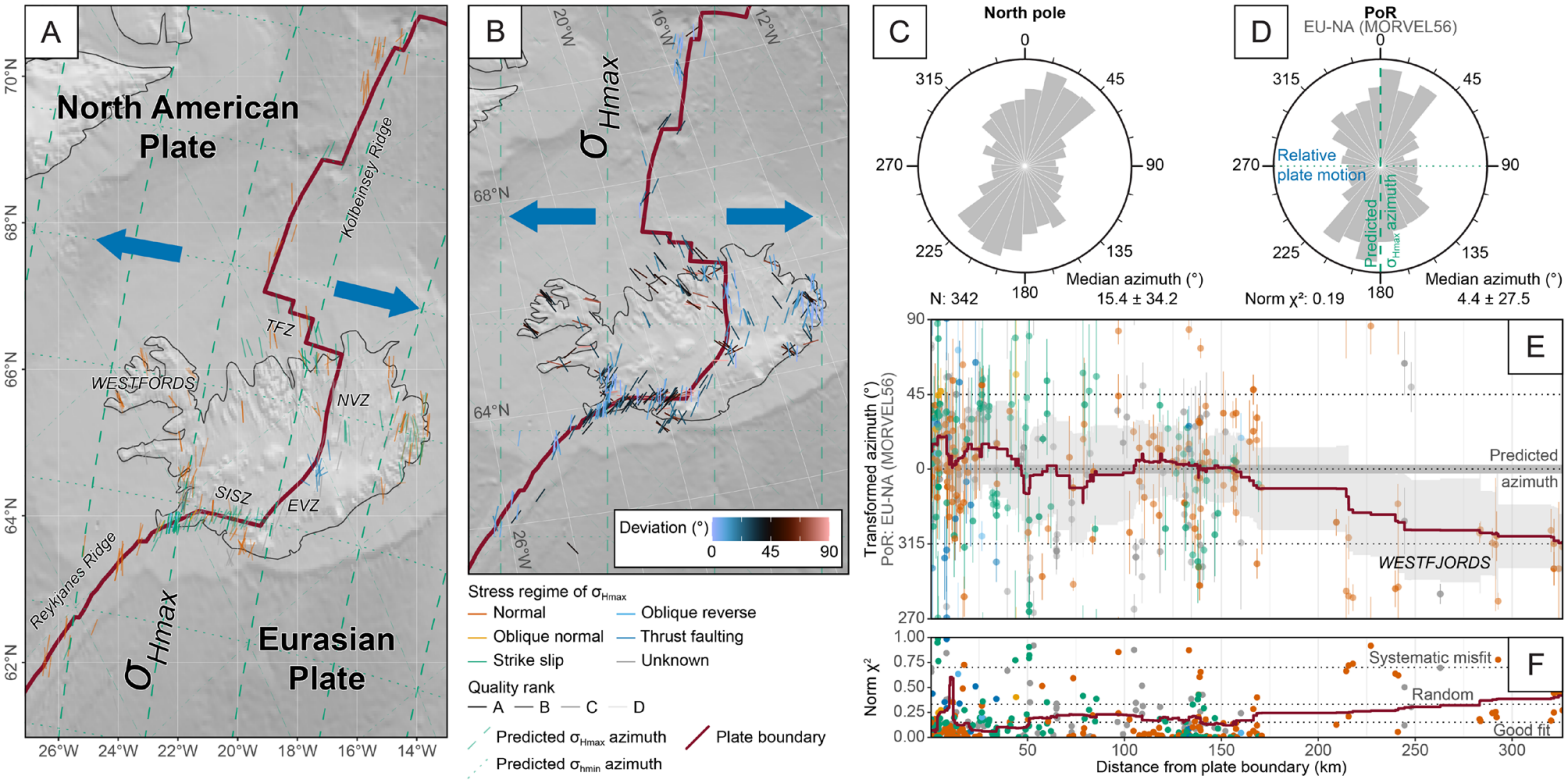
Stress field of the North Atlantic Ridge and Iceland. Stress trajectories are modeled from the relative plate motion between the North American (NA) and the Eurasian (EU) plates (based on NNR-MORVEL56). See Fig. 7 for details. Transform fracture zones: *SISZ* South Iceland Seismic Zone, *TFZ* Tjörnes Fracture Zone. Bin-width of rose diagrams: 13$^{\circ }$.

The oceanic Mid-Atlantic Ridge in the North Atlantic separates the North American from the Eurasian plate. Subaerial exposures of the ridge occur in Iceland where the oceanic ridge traverses a large-volume volcanic anomaly^89^. Here, the spreading rate ranges from 18 to 22 mm/yr^90^. The tectonic structures of Iceland are characterized by purely divergent rift segments located in the Northern Volcanic Zone in northern Iceland and the subparallel Western and Eastern Volcanic Zones in central Iceland (Fig. 11). These rifts are separated from the Atlantic Ridge by transform fracture zones. In the south, the sinistral South Iceland Seismic Zone connects the northern end of the Reykjanes Ridge and the Eastern Volcanic Zone^90,91^. In the north, the dextral Tjörnes Fracture Zone separates the Northern Volcanic Zone and the southern end of the Kolbeinsey Ridge (Fig. 11).

The stress field of the area is characterized by N-NNE trending $\sigma _\text {Hmax}$, generally parallel to the rift axis^90, 92^. Along the South Iceland Seismic Zone, $\sigma _\text {Hmax}$ trends NNE-NE. In the Westfjords, the oldest part of Iceland, $\sigma _\text {Hmax}$ rotates in an NNW-NW direction, approaching the direction of divergence^92^.

We test the stress field against an outward-moving plate boundary (Fig. 3) because of the divergent character of the plate boundary between North America and Eurasia. Therefore, $\sigma _\text {Hmax}$ is expected to be oriented perpendicular to relative plate motion (Fig. 4), i.e. parallel to great circles passing through the PoR associated with the relative plate motion (Fig. 5). The predicted orientation is 0$^{\circ }$ in the transformed CRS (Table 1). We expect a good fit of the $\sigma _\text {Hmax}$ directions adjacent to the rift axis. Misfits are expected farther away from the plate boundary and along the transform fault segments of the ridge.

The transformed azimuth of $\sigma _\text {Hmax}$ for the entire studied area (weighted circular median $4.4 \pm 27.5^{\circ }$ ) is parallel to the predicted orientation of 0$^{\circ }$ (Table 1) indicating a generally good fit ($\text {Norm}~\chi ^2$: 0.19, Tab. 2). Based on $\text {Norm}~\chi ^2$ criteria, the predictions from all four relative plate motion models yield acceptable fits with $\text {Norm}~\chi ^2$ $\le 0.202$ (Table 2). Thus, the test confirms the alignment of the $\sigma _\text {Hmax}$ orientation with the strike of the rift axis and the predicted orientation according to an outward-moving plate boundary (Fig. 11).

**Figure 12:**
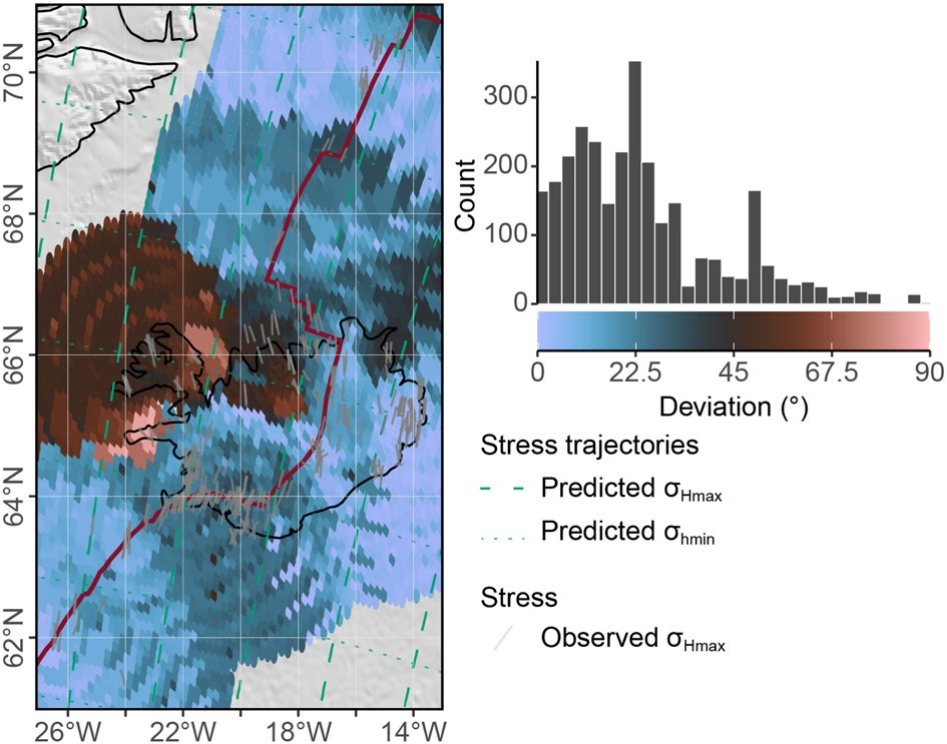
Deviation of the direction of the smoothed stress field from the predicted first-order stress field of the North Atlantic and Iceland. See Fig. 8 for interpolation parameters and map details.

Adjacent to the South Iceland Shear Zone and the Tjörnes Fracture Zone, however, the orientations of $\sigma _\text {Hmax}$ with predominantly strike-slip regimes deviate by ±45$^{\circ }$ from the predicted orientation indicating that $\sigma _\text {Hmax}$ follows clockwise and counterclockwise loxodromes passing through the PoR (Table 1), respectively. The spatial interpolation of the stress field reveals considerable deviations in the Westfjords of western Iceland (Fig. 12, the interpolated stress field is shown in Supplementary Fig. S1 online). The stress anomaly is described by the rotation of $\sigma _\text {Hmax}$ into the direction of divergence with increasing distance from the rift^45,58,92^. This stress rotation suggests the growing inward-moving displacement, and hence, the contribution of ridge push to the stress field.

### Global plate boundaries

**Figure 13:**
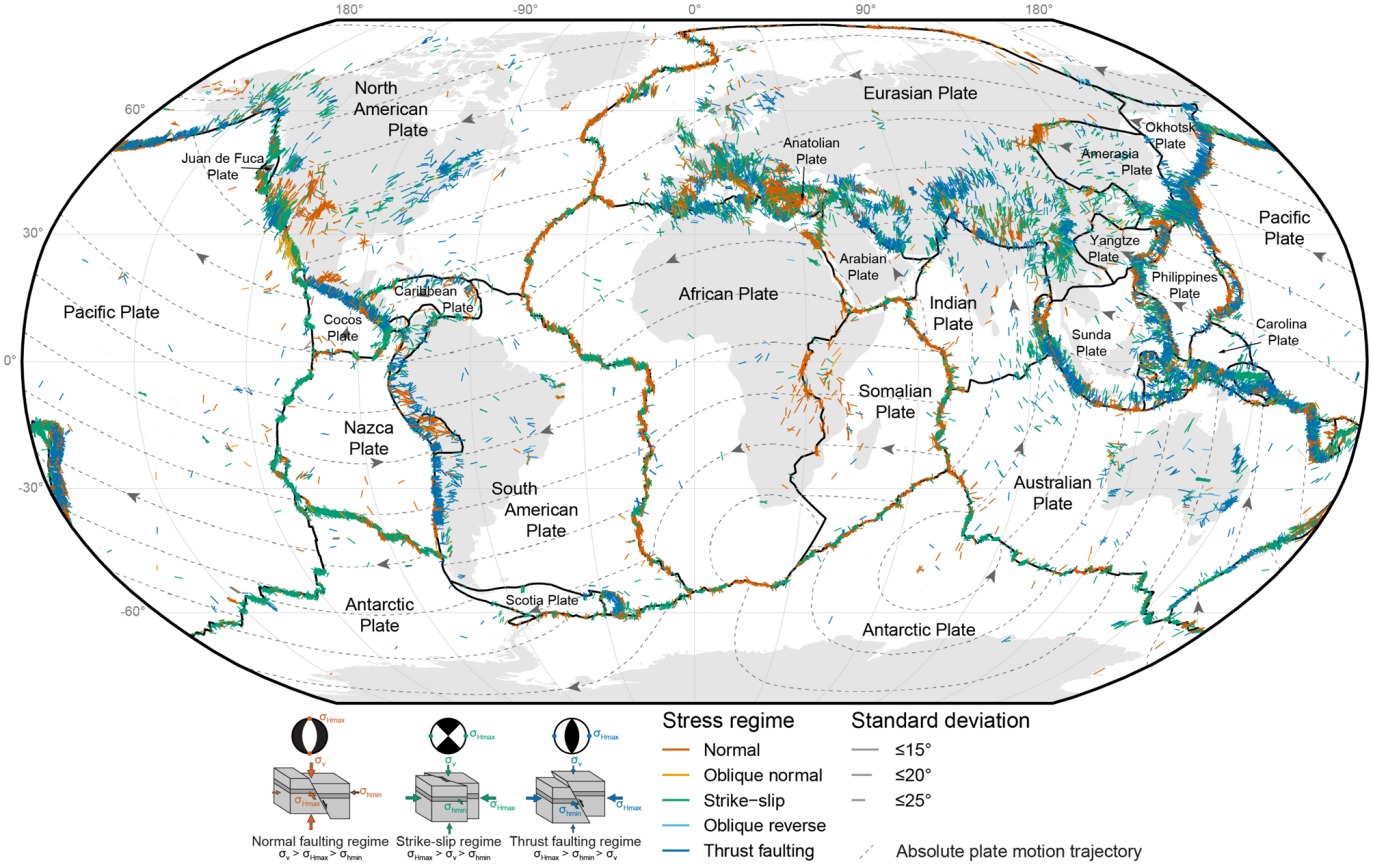
Global compilation of stress data showing the direction of the maximum horizontal stress ($\sigma _\text {Hmax}$) from the WSM2016 database (Robinson projection). The orientation of $\sigma _\text {Hmax}$ with respect to focal earthquake mechanisms and the three Andersonian fault types^93^ is shown. Data sources: plate boundaries^64^, absolute plate motion^54,65^ (hotspot reference frame).

In this last example for demonstrating our stress-field analysis we use the global set of WSM2016 stresses adjacent to plate boundaries. We consider only data points that are less than 1500 km and 500 km away from the associated convergent and divergent/transform plate boundaries, respectively (Fig. 13). A 500 or 1500 km buffer is created around the plate boundary to select these stress data points. Because buffering produces overlapping areas, in particular at triple junctions, the data points are assigned to the closest plate boundary using Eqs. (16) and (17). For statistical reasons, we omit plate boundaries that contain less than 10 data points on their perimeter. Divergent boundaries are often characterized by transfer zones crosscutting the rifts and, thus, strike-slip faulting occurs at short distances to normal faulting (Fig. 13). For that reason, we test stresses with strike-slip and normal fault regimes against the predicted $\sigma _\text {Hmax}$ orientation associated with tangentially and outward-moving plate boundaries, respectively. The plate motion parameters used for prediction are extracted from the GSRM v2.1 model.

**Figure 14:**
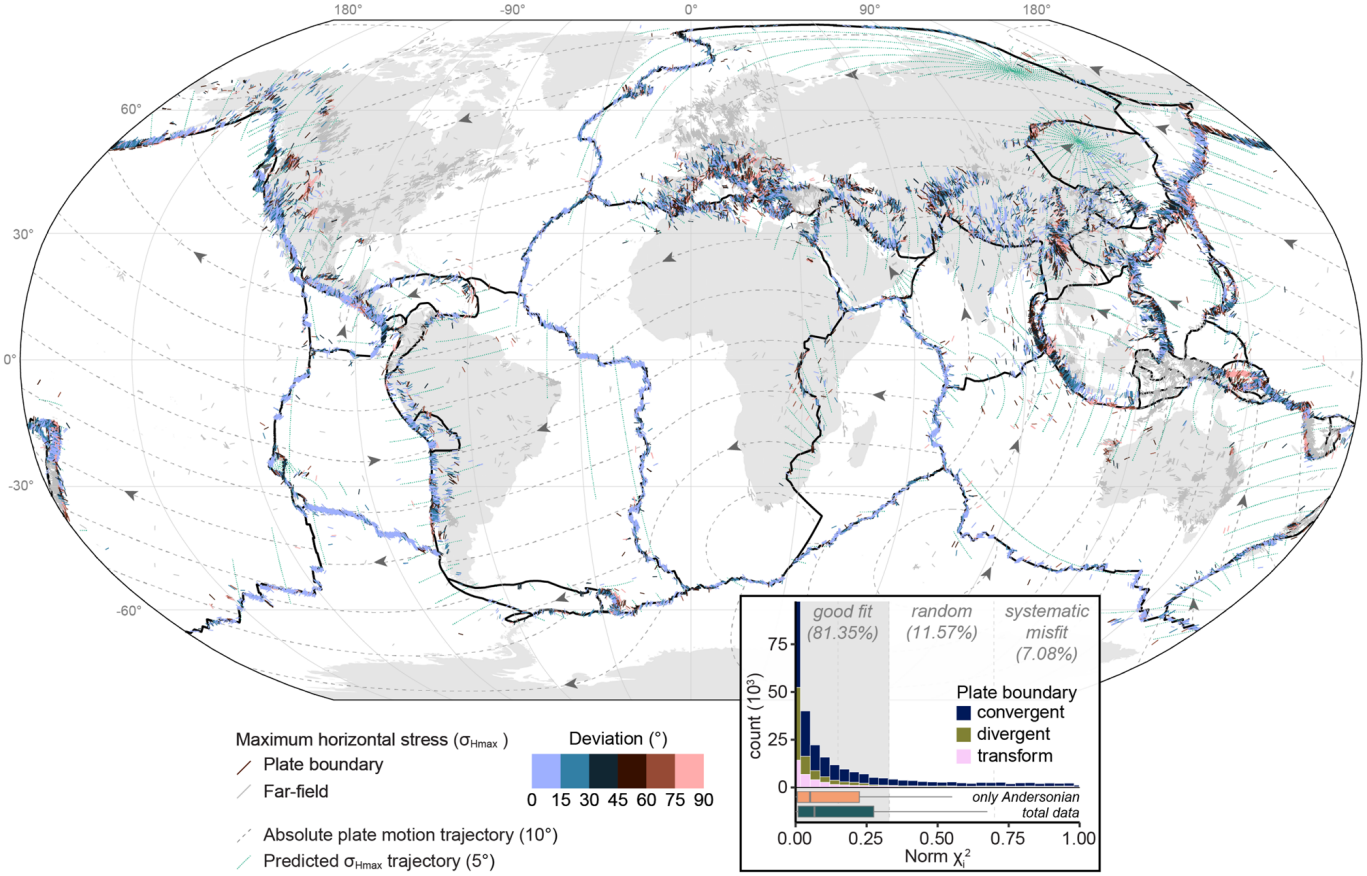
Deviation of global plate boundary stress fields from the predicted orientation of plate boundary forces. Each data point represents the orientation of a maximum horizontal stress ($\sigma _\text {Hmax}$) measurement from the WSM2016 database (only Andersonian states of stress are considered) and the color depicts the model misfit. The misfit is the deviation of the azimuth of $\sigma _\text {Hmax}$ from the predicted stress. The predicted stress is deduced from the relative plate motion parameters extracted from GSRM v2.1. See Fig. 13 for details. Inset shows the distribution of the $\text {Norm}~\chi ^2$ statistics that reflect the goodness-of-fit for the predicted orientation of the plate boundary stress with respect to the observed orientation of $\sigma _\text {Hmax}$. Boxplots compare the distributions of the dataset including only Andersonian states of stresses with the complete WSM2016 dataset.

The entire list of the results of our analysis of the global WSM2016 data (n = 33,081) can be found as Supplementary Table S1 online. Figure 14 depicts the results and the statistical agreement between the observed and predicted orientations of $\sigma _\text {Hmax}$ adjacent to plate boundaries. Using the $\text {Norm}~\chi ^2$ criterion, 64% of the stresses show good agreement with the predicted $\sigma _\text {Hmax}$ orientation. Another 15% are in the acceptable range, 14% are randomly distributed, and 8% show a systematic misfit to the prediction. Thus, ca. 79% of the global data statistically correlate with the predicted $\sigma _\text {Hmax}$ orientation of the theory. Considering only Andersonian stresses^93^ (n = 27,394), i.e. omitting oblique stress regimes such as oblique normal faults or oblique thrusts, yields a better fit. Thereby, 81% of pure thrusts, normal faults, and strike-slip faults are statistically parallel to the predicted $\sigma _\text {Hmax}$ direction (Fig. 13).

The overall good fit (Fig. 14) highlights that the stress field of the plate boundary zone can be satisfactorily explained by horizontal forces acting on lateral plate boundaries in the direction of the relative motion between the neighboring plates. The best-fitting plate boundaries are divergent plate boundaries where the $\sigma _\text {Hmax}$ orientation of 92% of the data fit the predicted orientation. Adjacent to transform and convergent plate boundaries ca. 81% and 77% of the data points, respectively, show a statistically good fit (Fig. 14). The stress-field analysis also identifies areas and tectonic settings where the predicted orientation of $\sigma _\text {Hmax}$ considerably deviates from the observed stress direction. These include the convergent plate boundaries in Europe, western North America, East Asia, and others (Fig. 14).

## Discussion

### Significance of the predicted stress direction

According to the presented theory of intraplate stresses, the only control on the orientation of the first-order stress is the relative motion of the plate. Thus, the precision of the stress analysis depends on the uncertainties of the observed stress data and the parameters for plate motion. Because any PoR has a positional uncertainty $\sigma (\text {PoR})$, the predicted orientation $\beta$ of $\sigma _\text {Hmax}$ will also be subjected to a certain error $\sigma (\beta )$. The maximum error of $\beta$ depends on the distance $\gamma$ of the data point from the PoR (Eq. 9). The maximum error of the predicted $\sigma _\text {Hmax}$ direction caused by the precision of the PoR is described by Ramsay^94^, p. 14:

18 $$\begin{aligned} \text {max}~\sigma (\beta ) = \frac{1}{2} \arccos {\sqrt{1- \frac{2 \sin ^2{\sigma (\text {PoR})}}{\sin ^2{\gamma }} (1 + \cos {\gamma })}} \end{aligned}$$

**Figure 15:**
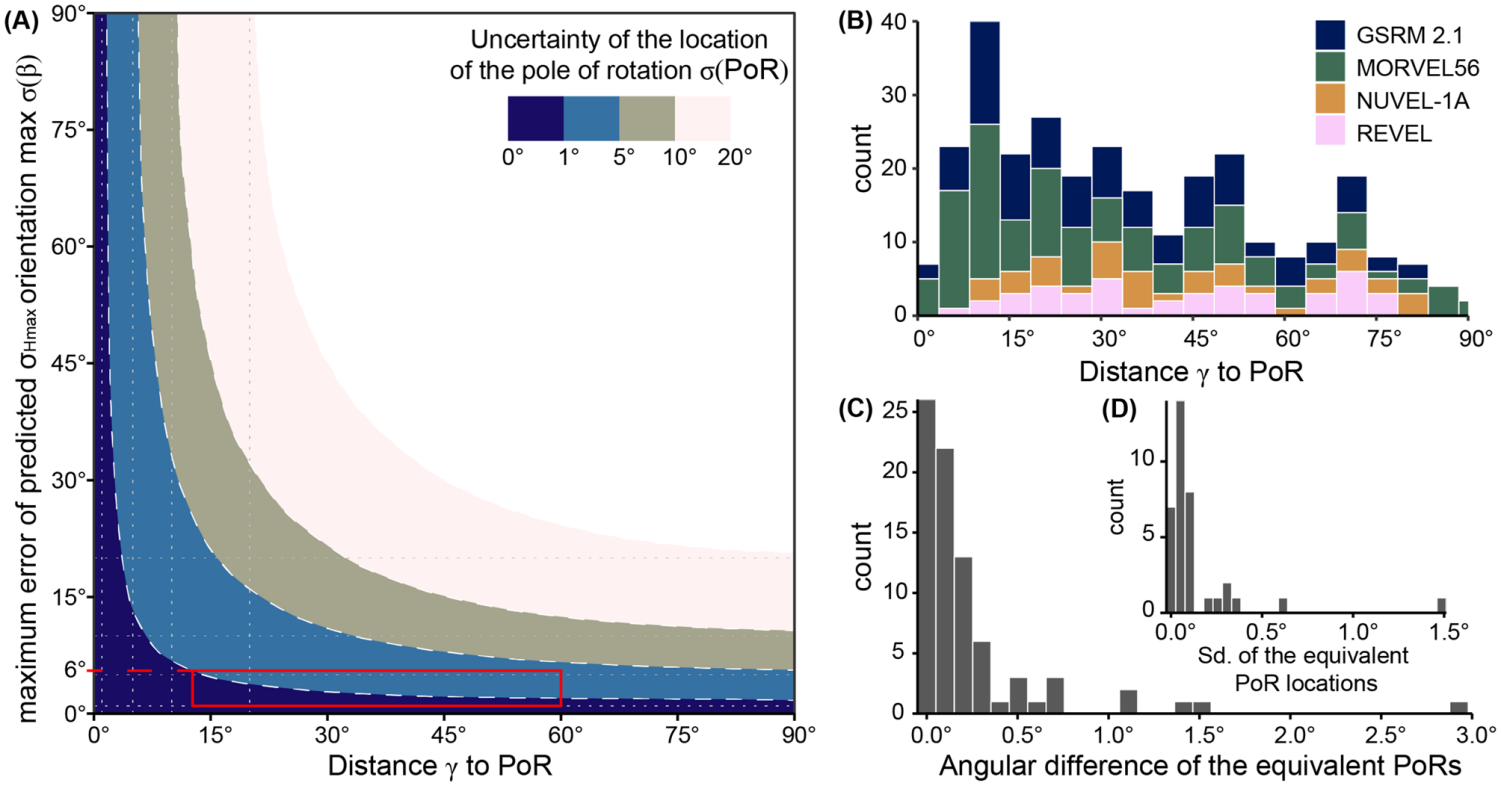
Influence of PoR uncertainty on the maximum error of the predicted $\sigma _\text {Hmax}$ orientation. (**A**) Isolines show maximum errors in predicting the stress direction depending on the distance to the PoR. Errors are shown for a synthetic stress data set (constant azimuth on all points on Earth) in the PoR coordinate reference system. Various uncertainties in the PoR location are simulated by shifting the pole 1, 5, 10, and 20$^{\circ }$ from the true position. Red rectangle marks the field of the global WSM2016 dataset, indicating that the maximum error for the predicted $\sigma _\text {Hmax}$ orientation does not exceed 6$^{\circ }$ on average. (**B**) Distribution of the distance of all plate boundary zones to their associated PoR for the relative motion of neighboring plates. (**C**) Distribution of the angular difference between equivalent PoRs from different plate motion models. Scattering is used to estimate the uncertainty of the PoR location. (**D**) Distribution of the standard deviation (Sd.) of the locations of equivalent PoRs from different plate motion models.

The values of this maximum error for various uncertainties of the PoR ($\sigma (\text {PoR})$) and the distance to the PoR are illustrated in Fig. 15A. The deviation of the predicted and observed $\sigma _\text {Hmax}$ may become as large as 90$^{\circ }$ for data located close to the PoR. In contrast, at the PoR’s equator, the maximum error of the predicted $\sigma _\text {Hmax}$ orientation is equal to the precision of the PoR’s position.

The uncertainties of the parameters for absolute plate motion can be large and differ considerably between the various global plate motion models. In contrast, the differences between the location of equivalent PoRs for relative plate motion are small between each model. PoRs are $<1^{\circ }$ away from their model equivalents (average standard deviation of pole distribution: 0.14$^{\circ }$, Fig. 15C and D). As shown for the areas of the San Andreas Fault–Gulf of California, Central Asia, and the North Atlantic Ridge–Iceland, the choice of the plate motion model does not have a considerable effect on the stress analysis because the different models yield similar results (Table 2). The general agreement between the parameters for the relative plate motions may suggest that the actual uncertainty of the PoRs might also be very small. Hence, it does not have a measurable effect on the accuracy of the predicted stress orientation.

To ensure that the error of the predicted $\sigma _\text {Hmax}$ orientation does not exceed an uncertainty of, e.g. $\le 25^{\circ }$ (C-quality in the WSM2016 data) of the observed data, the stress data should have at least a distance of 5$^{\circ }$ from the PoR (Fig. 15). The test datasets from the San Andreas Fault–Gulf of California, Central Asia, and the North Atlantic Ridge–Iceland areas, have larger distances to the PoR, namely 47–67$^{\circ }$, 19–44$^{\circ }$, and 34–54$^{\circ }$, respectively (Table 2). The vast majority of the global dataset also is unlikely to be considerably biased by the PoR distance because the PoRs are in ca. 13–55$^{\circ }$ distance to the tested plate boundary zones (Fig. 15B).

The scattering of the PoR locations of the different plate motions for the same relative plate motion can be used to estimate the uncertainty of the PoR locations. For the tested areas, this estimation and the distribution of the distances of the plate boundary zones to the PoR yield $\text {max}~\sigma (\beta )$ values ranging between 0.8$^{\circ }$ and 6.4$^{\circ }$ (Table 2). For the global dataset, the maximum error of the predicted $\sigma _\text {Hmax}$ orientations is ca. 6$^{\circ }$. Thus, the estimated maximum error for the direction of $\sigma _\text {Hmax}$ predicted by relative plate motion is smaller than the uncertainties of the stress orientation in the WSM2016 dataset, indicating that the predictions are significant.

### Non-Andersonian stress regimes

Because the predicted stresses are assumed to be generated by only horizontal compression and/or extension from the lateral plate boundary forces, two principal stress axes should be horizontal. The relative magnitude of the vertical principal stress, thereby, determines the stress regime. This assumption reflects the Andersonian regimes of stresses^93^, i.e. $\sigma _\text {Hmax}$ are parallel to the axis of maximum principal stress, unless that axis is vertical, in which case it is parallel to the axis of intermediate stress (Fig. 13). The Andersonian model of stress, which derives from the inference that the Earth’s approximately flat surface supports no shear stresses, agrees with typical stress geometries observed in a variety of tectonic situations for the upper crust^95,96^, and references therein.

The assumption of our stress orientation theory implies that the $\sigma _\text {Hmax}$ orientation of non-Andersonian stresses (no vertical principal stress axes) cannot be predicted by the model of Wdowinski^16^. In other words, these non-Andersonian stresses may deviate from the predicted $\sigma _\text {Hmax}$ orientation. Indeed, the predicted orientation from our theory for stress fields on the global stress dataset reveals a better fit for a dataset that does not consider non-Andersonian stresses (Fig. 14). The difference in the overall goodness-of-fit to the full dataset is, however, small. As shown for the San Andreas Faul–Gulf of California region, the $\sigma _\text {Hmax}$ orientation of non-Andersonian stresses only slightly deviates from the predicted first-order stress orientation (Fig. 7). There might be some additional bias in the WSM2016 data because the plunge of $\sigma _\text {Hmax}$ is not necessarily horizontal in the database. The reported $\sigma _\text {Hmax}$ orientation represents an approximation using the azimuth of the larger subhorizontal principal stress^17,32^. Hence this proxy can deviate from the true orientation by several ten degrees (for discussion see Ref.^97^). A more detailed analysis of the effect of non-Andersonian stresses on the results of stress-field analyses is required. However, this is beyond the scope of this study as the exact orientation of the principal axes is not documented in the used WSM2016 dataset.

### Stress anomalies

Our proposed method for stress-field analysis and data interpolation allows mapping of the goodness-of-fit between observed and predicted stress orientations. Identified regions of systematic misfit are those where the lateral plate boundary force does not control the $\sigma _\text {Hmax}$ orientation. Stress deviations are generally associated with the superposition of the first-order stress field with local stresses that may originate from buoyancy gradients. These gradients may be caused by dynamic topography, lithospheric flexure, deglaciation, or smaller-scale lateral density contrasts within the crust^3,32,64,98–101^. Our example from Iceland, for instance, shows that the stress anomaly in western Iceland reflects the rotation of the stress from plate boundary parallel to perpendicular directions with increasing distance to the plate boundary (Fig. 12). The rotation is explained to be caused by the increasing contribution of additional far-field stresses, such as ridge push causing inward-displacement^92^. Lateral escape motions due to the gravitational collapse of overthickened crust in the Tibetan Plateau^81–83^ are interpreted to generate large stress anomalies to the east and the west of Tibet (Fig. 10).

The example from the San Andreas Faul–Gulf of California region shows substantial stress deviations for the Colorado Plateau (Fig. 8). Here, the high spatial resolution (0.25$^{\circ }$ grid size) of the modeled stress field outlines this area on a spatial scale that can be compared with geological features that may cause additional stresses. Similarly, but on a much larger scale, our global stress-field analysis reveals plate boundary zones with substantial deviations between observed and predicted stress orientations (Fig. 14). These regions are notably found near convergent plate boundaries, such as in western North America, southern Europe, or East Asia (Figs. 10 and 14). It is worth noting that the model by Wdowinski^16^ assumes homogeneous stress within a mechanically isotropic crust. That means the proposed theory for stress orientations can be used to identify regions where the crust is characterized by strong lithological and structural heterogeneities^4,102–111^. Regions of lateral heterogeneities are particularly expected on continental plates that preserve a much longer geological history than oceanic plates. For that reason, we see a much better fit between observed and predicted $\sigma _\text {Hmax}$ orientations along spreading ridges than along convergent plate boundaries that involve one or two lithospheric domains (Figs. 12 and 14).

## Conclusions

The geometry of a stress field depends on the underlying coordinate reference system in which the data is analyzed (Fig. 1). In the geographical coordinate reference system, the meaning of a spatially uniform (or homogeneous) stress field is difficult to extract and likely does not exist. Our updated theory for intraplate stress allows a more robust definition of a spatially uniform stress field. Because stress fields are analyzed from the perspective of its first-order stress source (e.g. plate boundary forces). Here, uniformity is defined by stress orientations parallel to plate boundary forces.

This approach provides a powerful technique for analyzing and predicting stress fields based on the empirical correlation between lateral plate boundary forces and the first-order orientation of stresses^16^. Using simple assumptions, the updated model for stress-field orientations only requires the well constrained parameters for relative plate motion. The benefits are: (i) the predictions of the model do not depend on the sample size, (ii) the robust stress analysis and predictions do not suffer from angle distortions caused by the curvature of the Earth, (iii) the proposed technique can be combined with other stress interpolation methods, and (iv) it is not limited to the analysis of stress orientation data and can be applied to any orientation data measured at discrete locations (e.g. strain data, mineral or intersection lineations, and lineaments).

The preservation of angles allows thorough orientation analysis of large-scale tectonic structures, such as faults and folds (Figs. 7B, 9B, and 11B). It is, therefore, recommended that any stress-field analysis, interpolation of $\sigma _\text {Hmax}$ orientations, and map-based analysis of young deformation structures such as faults and folds should be performed in the PoR CRS, particularly in areas close to a plate boundary.

We show that the orientation of more than 80% of the global stress data adjacent to plate boundaries is parallel to our model predictions (Fig. 14). That means the majority of the stress data can be sufficiently explained by plate boundary forces. This result emphasizes the importance of plate boundary forces for lithospheric stress and strain. Because plate boundary forces can be transferred far inboard ($\ge$3000 km, Fig. 9), the technique also allows for analyzing far-field stresses and the superposition of various stress fields. Deviations from the modeled first-order stress field reveal the presence of second-order stress fields and allow evaluation of other stress sources. The identification of such stress anomalies can guide targeted future studies. Moreover, the azimuth and coordinate transformation is helpful for interpreting the orientation of young large-scale tectonic structures because the geometries are not biased by angular distortion.

The proposed model for analyzing stress fields is particularly useful in regions where stress information is rare or instrumental earthquake recordings are unavailable. Thus, it can be used to estimate fault activity through additional kinematic or dynamic models and predict the stress orientations of future earthquakes. For immediate use, the model’s algorithms are implemented in the free and open-source software package tectonicr.

### Supplementary Information

Supplementary Information 1.

Supplementary Information 2.

## Data Availability

The datasets analyzed during the current study are available in the World Stress Map database release 2016 repository, https://doi.org/10.5880/WSM.2016.001. The software used for the analysis and for reproduction of the figures is available in the Zenodo repository, https://doi.org/10.5281/zenodo.7510800.
