# Supplementary material for: Analyzing the horizontal orientation of the crustal stress adjacent to plate boundaries
Source: Sci Rep. 2023 Sep 20;13:15590. doi: 10.1038/s41598-023-42433-2 (PMC10511519; doi:10.1038/s41598-023-42433-2)
Supplement: Supplementary file 1 — Supplementary Information 1. [file 41598_2023_42433_MOESM1_ESM.pdf]

# Analyzing the horizontal orientation of the crustal stress adjacent to plate boundaries:
# Supplementary Information

Tobias Stephan, Eva Enkelmann, Uwe Kroner

April 27, 2023

## 1 Conversion between geographical and Cartesian coordinates

The coordinate conversion of a vector given in geographical coordinates (latitude $\lambda$ and longitude $\phi$) into Cartesian coordinates is

$$
\begin{aligned}
x &= \cos\lambda \cos\phi \\
y &= \cos\lambda \cos\phi \\
z &= \sin\lambda
\end{aligned}
$$

The conversion into geographical coordinates is

$$
\begin{aligned}
\lambda &= \arcsin\left(\frac{z}{\sqrt{x^2+y^2+z^2}}\right) \\
\phi &= \operatorname{arctan2}(y,\, x)
\end{aligned}
$$

## 2 Implementation in R

The presented method and the example dataset are implemented in the free and open-source software package `tectonicr` that is written in the computer language R. As a R-based cross-platform program, `tectonicr` works on Windows©, MacOS X© and GNU/Linux. The source code for the program is made available under the GNU GPL license v3.0, which permits re-use and modification provided that any derived code is released under the same conditions (Free Software Foundation, 2007). The entire source code of `tectonicr` is accessible through the Comprehensive R Archive Network (CRAN)[1] and can be accessed through R's command line:

```
install.packages("tectonicr")
library("tectonicr")
```

The latest version can be obtained from the GitHub repository (`https://github.com/tobiste/tectonicr`) requiring the package `remotes`:

```
install.packages("remotes")
remotes::install_github("tobiste/tectonicr")
library("tectonicr")
```

The following code reproduces the examples used in the study. It transforms the San Andreas Fault-Gulf of California stress dataset into the coordinate reference system of the North America-Pacific plate motion, evaluates the transformed stress orientations in terms of a right-lateral plate boundary displacement, and visualizes the results:

```
# Load the San Andreas-Gulf of California dataset:
data("san_andreas")
# Load plate boundary geometries
data("plates")
```

[1] The software will be uploaded to CRAN once the review is completed. Meanwhile, a beta-version is available through the GitHub repository.

```r
# Extract boundary between Pacific (pa) and North American (na) plates
na_pa_boundary <- subset(plates, plates$pair == "na-pa")
# Load the current plate motion (cpm) models:
data("cpm_models")
morvel <- subset(cpm_models, cpm_models$model == "NNR-MORVEL56") # select MORVEL model
# Relative plate motion between Pacific and North American plates:
na_pa <- equivalent_rotation(morvel, fixed = "na", rot = "pa")
# Transform stress data set and test against predicted left-lateral tangential plate
    boundary (left):
stress_analysis(san_andreas, euler = na_pa, type = "right", pb = na_pa_boundary)
# Interpolate the stress field in the PoR coordinate system:
PoR_stress2grid(san_andreas, na_pa)
```

The stress data used for the Himalaya-Tibet and North Atlantic Ridge-Iceland areas can be obtained and processed through the following code:

```r
data("tibet")
eu_in_boundary <- subset(plates, plates$pair == "eu-in")
eu_in <- equivalent_rotation(morvel, fixed = "eu", rot = "in")
stress_analysis(tibet, euler = eu_in, type = "in", pb = eu_in_boundary)
PoR_stress2grid(tibet, eu_in)

data("iceland")
eu_na_boundary <- subset(plates, plates$pair == "eu-na")
eu_na <- equivalent_rotation(morvel, fixed = "na", rot = "eu")
stress_analysis(iceland, euler = eu_na, type = "out", pb = eu_na_boundary)
PoR_stress2grid(iceland, eu_na)
```

## 3 Test results and spatial interpolation

`san_andreas.csv` Dataset of the San Andreas Fault-Gulf of California region (snippet from the WSM2016 database, table column headers are identical to the database).

`san_andreas_smooth.csv` Smoothed stress field for the San Andreas Fault-Gulf of California region as comma-separated text file (as shown in Supplementary Figure S1A).

`san_andreas_smooth_shp.zip` Smoothed stress field for the San Andreas Fault-Gulf of California region as ESRI shapefile.

`asia.csv` Dataset of Central Asia (snippet from the WSM2016 database)

`tibet_smooth.csv` Smoothed stress field for the Himalaya-Tibet region as comma-separated text file (as shown in Supplementary Figure S1B).

`tibet_smooth_shp.zip` Smoothed stress field for the Himalaya-Tibet region as ESRI shapefile.

`iceland.csv` Dataset of the North Atlantic Ridge-Iceland region (snippet from the WSM2016 database)

`iceland_smooth.csv` Smoothed stress field for the North Atlantic Ridge-Iceland region as comma-separated text file (as shown in Supplementary Figure S1C).

`iceland_smooth_shp.zip` Smoothed stress field for the North Atlantic Ridge-Iceland region as ESRI shapefile.

Datasets used in this study are extracted from the WSM2016 database. Hence, table columns of the files are named as in the database. The generated datasets contain the additional columns:

**azi_PoR, azi_PoR_predicted** Transformed observed azimuth and predicted azimuth of $\sigma_{\mathrm{Hmax}}$

**deviation** Deviation of observed azimuth from predicted azimuth

**lat_PoR, lon_PoR** PoR CRS transformed latitude and longitude

**distance_pb_deg, distance_pb_km** Distance to plate boundary given in degrees and km

**beta** Angle between strike of plate boundary and $\sigma_{\mathrm{Hmax}}$ azimuth

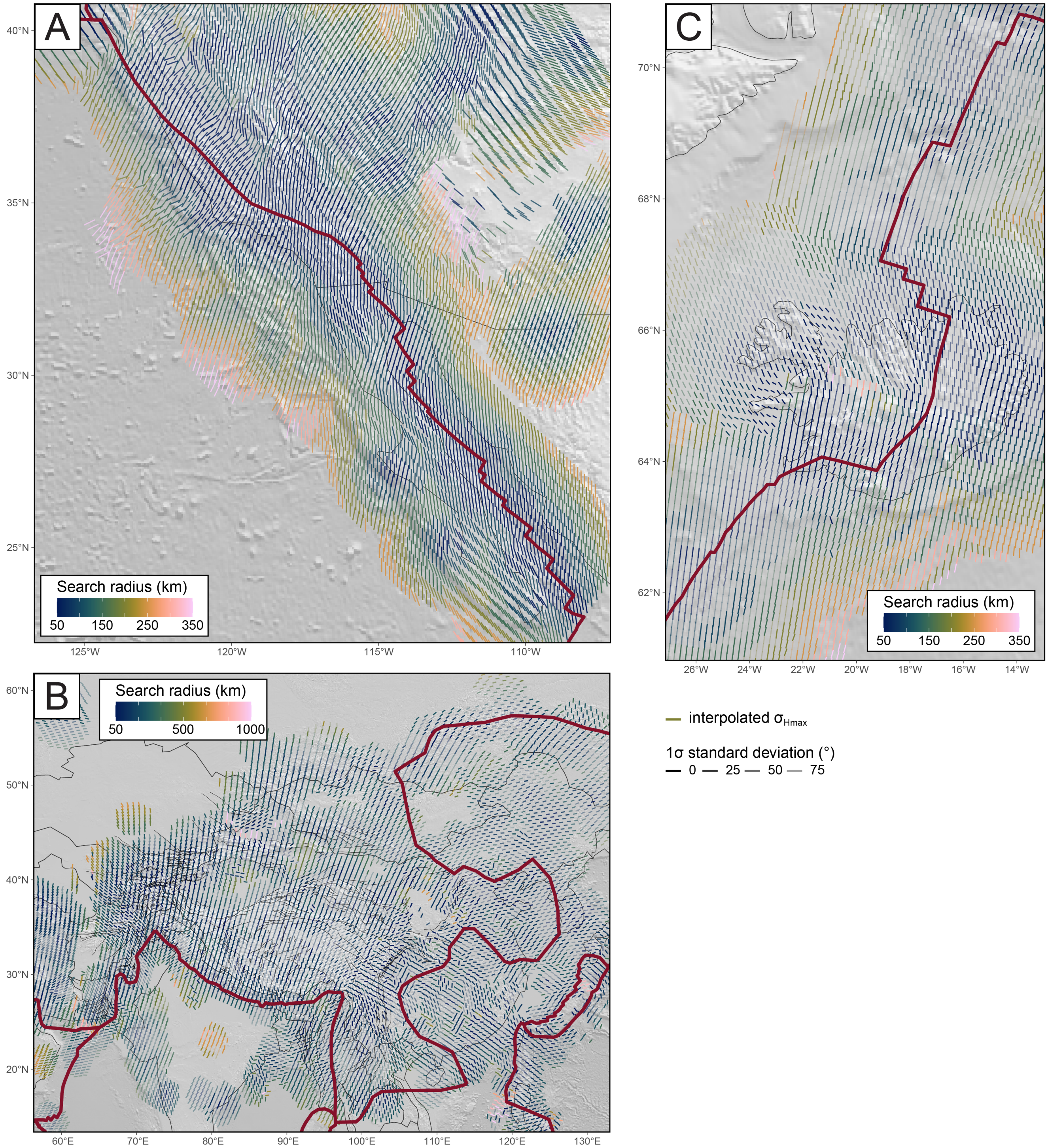


**Figure S1.** Interpolated stress fields. (A) San Andreas-Gulf of California (search radius: 50–350 km, grid size: 0.25°). (B) Central Asia (search radius: 50–1000 km, grid size: 0.25°). (C) North Atlantic Ridge-Iceland (search radius: 50–350 km, grid size: 0.125°). Plate boundary are depicted as red lines. Geographical coordinate reference system (Mercator projection). The shaded relief is based on ETOPO1. Maps are created with the R package `ggplot2`.

## 4 Test results and statistical summary for global set of plate boundaries

`plate_boundaries.zip` Geometries of the plate boundaries (from Bird 2003) with characterization of the plate boundary displacement type (ESRI shapefile).

`global_gsrm.csv` Test results for the global plate boundaries using GSRM v2.1

`global_morvel.csv` Test results for the global plate boundaries using MORVEL56

`global_nuvel.csv` Test results for the global plate boundaries using NUVEL-1A

`global_revel.csv` Test results for the global plate boundaries using REVEL

Datasets used for the interpolation are extracted from the WSM2016 database. Hence, table columns of the files are named as in the database. The generated datasets contain the additional columns as described in Section 3

**Table S1.** Statistical summary of testing global plate boundary zones ($\leq$1500 km from plate boundary) using different models for current plate motion. Transform plate boundaries are either right-lateral (R) or left-lateral (L). The observed azimuth is the weighted circular median and the circular quasi-interquartile range of the PoR-transformed $\sigma_{Hmax}$ azimuths.

| | | Azimuth wrt. PoR (°) | | Norm | Width | Distance to PoR |
|---|---|---|---|---|---|---|
| Type | Model | predicted | observed | $\chi^2$ | D (°) | $\gamma$ (°) |
| *Africa-Aegean Sea* | | | | | | |
| convergent | GSRM v2.1 | 90 | 89.8 ± 31.6 | 0.366 | 1.6 | 56 – 77 |
| | MORVEL56 | 90 | 86.1 ± 34.9 | 0.371 | 1.7 | 60 – 84 |
| *Africa-Eurasia* | | | | | | |
| convergent | GSRM v2.1 | 90 | 108.5 ± 29.8 | 0.229 | 6.8 | 34 – 75 |
| | MORVEL56 | 90 | 100.8 ± 27.2 | 0.264 | 10.8 | 6 – 48 |
| | NUVEL-1A | 90 | 99.3 ± 31.9 | 0.263 | 9.3 | 7 – 51 |
| | REVEL | 90 | 114.8 ± 36.2 | 0.197 | 6.4 | 45 – 79 |
| transform (R) | GSRM v2.1 | 135 | 116.3 ± 18.6 | 0.131 | 1.7 | 42 – 50 |
| | MORVEL56 | 135 | 106.5 ± 23.9 | 0.298 | 2.0 | 14 – 23 |
| | NUVEL-1A | 135 | 107.2 ± 23.7 | 0.295 | 2.0 | 14 – 23 |
| | REVEL | 135 | 125.9 ± 19.8 | 0.138 | 1.9 | 51 – 60 |
| *Amur-Okhotsk* | | | | | | |
| convergent | GSRM v2.1 | 90 | 104.3 ± 18.7 | 0.138 | 9.3 | 16 – 38 |
| | MORVEL56 | 90 | 129.8 ± 20.6 | 0.245 | 21.1 | 1 – 21 |
| | REVEL | 90 | 78.0 ± 25.9 | 0.210 | 10.2 | 4 – 29 |
| *Amur-Philippine Sea* | | | | | | |
| convergent | GSRM v2.1 | 90 | 93.9 ± 18.5 | 0.122 | 2.0 | 28 – 45 |
| | MORVEL56 | 90 | 89.6 ± 22.9 | 0.138 | 3.5 | 20 – 42 |
| *Antarctica-Scotia* | | | | | | |
| convergent | GSRM v2.1 | 90 | 113.8 ± 23.6 | 0.170 | 3.0 | 20 – 41 |
| | MORVEL56 | 90 | 74.9 ± 30.6 | 0.202 | 2.7 | 11 – 31 |
| | NUVEL-1A | 90 | 73.9 ± 20.8 | 0.098 | 3.5 | 13 – 34 |
| transform (L) | GSRM v2.1 | 45 | 76.3 ± 25.1 | 0.314 | 0.3 | 14 – 21 |
| | MORVEL56 | 45 | 34.3 ± 14.5 | 0.103 | 0.4 | 14 – 22 |
| | NUVEL-1A | 45 | 40.3 ± 21.6 | 0.132 | 0.3 | 15 – 21 |
| *Arabia-Eurasia* | | | | | | |
| convergent | GSRM v2.1 | 90 | 103.8 ± 25.4 | 0.489 | 7.9 | 26 – 49 |
| | MORVEL56 | 90 | 146.4 ± 26.4 | 0.575 | 7.6 | 26 – 45 |
| | NUVEL-1A | 90 | 108.4 ± 26.3 | 0.385 | 9.7 | 23 – 45 |
| | REVEL | 90 | 108.6 ± 29.0 | 0.291 | 11.4 | 17 – 35 |
| *Australia-Burma* | | | | | | |
| convergent | GSRM v2.1 | 90 | 49.9 ± 14.6 | 0.290 | 4.5 | 1 – 23 |
| | MORVEL56 | 90 | 65.5 ± 18.2 | 0.208 | 3.1 | 3 – 27 |
| *Australia-India* | | | | | | |

| | | | | | | |
|---|---|---|---|---|---|---|
| convergent | GSRM v2.1 | 90 | 97.4 ± 14.8 | 0.155 | 13.6 | 3 – 23 |
| | MORVEL56 | 90 | 101.8 ± 39.2 | 0.302 | 91.5 | 1 – 23 |
| | NUVEL-1A | 90 | 86.7 ± 39.3 | 0.278 | 86.8 | 3 – 26 |
| | REVEL | 90 | 83.3 ± 16.2 | 0.193 | 13.9 | 3 – 24 |
| *Australia-Pacific* | | | | | | |
| convergent | GSRM v2.1 | 90 | 79.1 ± 31.7 | 0.253 | 3.4 | 12 – 62 |
| | MORVEL56 | 90 | 76.7 ± 21.4 | 0.225 | 1.5 | 12 – 62 |
| | NUVEL-1A | 90 | 71.4 ± 29.0 | 0.107 | 3.3 | 11 – 67 |
| | REVEL | 90 | 72.5 ± 28.2 | 0.100 | 3.0 | 13 – 69 |
| transform (L) | GSRM v2.1 | 45 | 38.0 ± 12.0 | 0.063 | 1.1 | 41 – 47 |
| | MORVEL56 | 45 | 30.7 ± 9.5 | 0.047 | 0.7 | 43 – 45 |
| | NUVEL-1A | 45 | 20.3 ± 18.2 | 0.110 | 1.2 | 41 – 46 |
| | REVEL | 45 | 33.4 ± 17.9 | 0.099 | 1.2 | 42 – 48 |
| transform (R) | GSRM v2.1 | 135 | 123.8 ± 19.8 | 0.549 | 1.2 | 9 – 23 |
| | MORVEL56 | 135 | 127.8 ± 19.9 | 0.570 | 1.2 | 10 – 23 |
| | NUVEL-1A | 135 | 122.9 ± 19.9 | 0.539 | 1.2 | 8 – 23 |
| | REVEL | 135 | 124.3 ± 19.3 | 0.561 | 1.2 | 10 – 25 |
| *Australia-Sunda* | | | | | | |
| convergent | GSRM v2.1 | 90 | 110.6 ± 33.6 | 0.448 | 3.0 | 40 – 85 |
| | MORVEL56 | 90 | 113.7 ± 25.2 | 0.456 | 2.8 | 40 – 85 |
| | REVEL | 90 | 109.8 ± 28.7 | 0.484 | 3.5 | 35 – 79 |
| *Australia-Woodlark* | | | | | | |
| convergent | GSRM v2.1 | 90 | 64.4 ± 21.1 | 0.413 | 47.4 | 2 – 13 |
| | MORVEL56 | 90 | 18.2 ± 33.7 | 0.509 | 140.1 | 2 – 12 |
| divergent | GSRM v2.1 | 0 | 137.5 ± 21.7 | 0.159 | 1.9 | 5 – 10 |
| | MORVEL56 | 0 | 140.8 ± 15.0 | 0.190 | 2.0 | 3 – 11 |
| *Burma-Eurasia* | | | | | | |
| convergent | GSRM v2.1 | 90 | 127.6 ± 22.6 | 0.347 | 12.5 | 10 – 13 |
| | MORVEL56 | 90 | 136.8 ± 25.2 | 0.393 | 13.4 | 10 – 11 |
| *Burma-India* | | | | | | |
| convergent | GSRM v2.1 | 90 | 165.2 ± 44.5 | 0.276 | 4.4 | 3 – 17 |
| | MORVEL56 | 90 | 73.3 ± 42.6 | 0.326 | 7.2 | 3 – 23 |
| *Caribbean-Cocos* | | | | | | |
| convergent | GSRM v2.1 | 90 | 97.8 ± 13.3 | 0.152 | 1.6 | 38 – 54 |
| | MORVEL56 | 90 | 97.0 ± 18.3 | 0.156 | 1.8 | 38 – 52 |
| | NUVEL-1A | 90 | 92.4 ± 11.9 | 0.173 | 2.2 | 26 – 43 |
| *Caribbean-South America* | | | | | | |
| convergent | GSRM v2.1 | 90 | 2.4 ± 27.5 | 0.589 | 3.9 | 43 – 64 |
| | MORVEL56 | 90 | 6.2 ± 26.1 | 0.656 | 3.0 | 40 – 61 |
| | NUVEL-1A | 90 | 175.7 ± 24.4 | 0.562 | 5.0 | 32 – 53 |
| | REVEL | 90 | 176.1 ± 24.5 | 0.551 | 4.7 | 35 – 56 |
| transform (R) | GSRM v2.1 | 135 | 137.7 ± 18.2 | 0.096 | 0.7 | 45 – 52 |
| | MORVEL56 | 135 | 140.9 ± 16.7 | 0.102 | 0.6 | 42 – 49 |
| | NUVEL-1A | 135 | 130.6 ± 19.4 | 0.074 | 0.7 | 36 – 42 |
| | REVEL | 135 | 131.5 ± 19.3 | 0.076 | 0.7 | 39 – 45 |
| *Caroline-Woodlark* | | | | | | |
| convergent | GSRM v2.1 | 90 | 71.6 ± 14.4 | 0.113 | 1.3 | 16 – 23 |
| | MORVEL56 | 90 | 69.7 ± 13.6 | 0.117 | 1.0 | 23 – 28 |
| *Cocos-North America* | | | | | | |
| convergent | GSRM v2.1 | 90 | 86.3 ± 24.8 | 0.365 | 9.4 | 22 – 52 |
| | MORVEL56 | 90 | 85.8 ± 24.3 | 0.363 | 9.4 | 22 – 53 |
| | NUVEL-1A | 90 | 83.7 ± 22.4 | 0.360 | 15.6 | 12 – 42 |
| *Cocos-Panama* | | | | | | |
| convergent | GSRM v2.1 | 90 | 106.4 ± 12.9 | 0.147 | 1.2 | 48 – 64 |
| | MORVEL56 | 90 | 97.0 ± 11.8 | 0.118 | 1.1 | 39 – 47 |

| | | | | | | |
|---|---|---|---|---|---|---|
| *Eurasia-India* | | | | | | |
| convergent | GSRM v2.1 | 90 | 87.7 ± 29.6 | 0.656 | 6.4 | 34 – 82 |
| | MORVEL56 | 90 | 8.9 ± 33.1 | 0.722 | 6.4 | 32 – 81 |
| | NUVEL-1A | 90 | 178.8 ± 33.8 | 0.642 | 6.3 | 35 – 83 |
| | REVEL | 90 | 8.3 ± 33.4 | 0.721 | 6.0 | 38 – 87 |
| transform (L) | GSRM v2.1 | 45 | 78.0 ± 23.7 | 0.234 | 1.3 | 41 – 51 |
| | MORVEL56 | 45 | 72.2 ± 23.4 | 0.202 | 1.3 | 40 – 50 |
| | NUVEL-1A | 45 | 81.0 ± 23.8 | 0.252 | 1.3 | 42 – 52 |
| | REVEL | 45 | 73.3 ± 23.6 | 0.210 | 1.3 | 46 – 55 |
| transform (R) | GSRM v2.1 | 135 | 113.2 ± 24.0 | 0.137 | 1.9 | 65 – 75 |
| | MORVEL56 | 135 | 109.2 ± 26.6 | 0.162 | 1.9 | 64 – 74 |
| | NUVEL-1A | 135 | 115.4 ± 23.2 | 0.180 | 1.9 | 66 – 76 |
| | REVEL | 135 | 111.0 ± 25.7 | 0.154 | 1.9 | 69 – 80 |
| *Eurasia-Sunda* | | | | | | |
| convergent | GSRM v2.1 | 90 | 13.0 ± 30.4 | 0.608 | 3.5 | 37 – 58 |
| | MORVEL56 | 90 | 178.2 ± 17.3 | 0.461 | 3.6 | 51 – 81 |
| | REVEL | 90 | 65.8 ± 41.0 | 0.379 | 26.3 | 12 – 34 |
| *Juan de Fuca-North America* | | | | | | |
| convergent | GSRM v2.1 | 90 | 54.6 ± 19.3 | 0.055 | 18.8 | 3 – 37 |
| | MORVEL56 | 90 | 55.7 ± 18.9 | 0.047 | 18.6 | 3 – 38 |
| | NUVEL-1A | 90 | 50.2 ± 19.3 | 0.122 | 12.5 | 12 – 46 |
| *Juan Fernandez-Nazca* | | | | | | |
| convergent | GSRM v2.1 | 90 | 80.1 ± 12.5 | 0.179 | 40.0 | 0 – 13 |
| | MORVEL56 | 90 | 90.3 ± 11.1 | 0.159 | 41.3 | 0 – 13 |
| *Mariana-Pacific* | | | | | | |
| convergent | GSRM v2.1 | 90 | 110.1 ± 36.2 | 0.276 | 4.2 | 6 – 18 |
| | MORVEL56 | 90 | 117.7 ± 34.9 | 0.294 | 2.0 | 3 – 30 |
| *North America-Okhotsk* | | | | | | |
| convergent | GSRM v2.1 | 90 | 38.1 ± 17.8 | 0.431 | 4.0 | 9 – 24 |
| | MORVEL56 | 90 | 157.2 ± 22.6 | 0.504 | 5.0 | 8 – 17 |
| | REVEL | 90 | 23.8 ± 23.6 | 0.518 | 7.8 | 9 – 26 |
| transform (R) | GSRM v2.1 | 135 | 155.3 ± 43.3 | 0.171 | 2.2 | 6 – 19 |
| | MORVEL56 | 135 | 169.8 ± 35.2 | 0.339 | 0.7 | 8 – 14 |
| | REVEL | 135 | 58.1 ± 22.2 | 0.554 | 2.0 | 3 – 13 |
| *North America-Pacific* | | | | | | |
| convergent | GSRM v2.1 | 90 | 105.0 ± 18.5 | 0.509 | 4.1 | 24 – 77 |
| | MORVEL56 | 90 | 106.9 ± 19.1 | 0.558 | 3.9 | 27 – 79 |
| | NUVEL-1A | 90 | 103.5 ± 19.4 | 0.462 | 4.2 | 23 – 77 |
| | REVEL | 90 | 109.3 ± 17.8 | 0.593 | 3.8 | 26 – 77 |
| transform (R) | GSRM v2.1 | 135 | 134.9 ± 14.4 | 0.022 | 0.7 | 29 – 40 |
| | MORVEL56 | 135 | 134.0 ± 14.0 | 0.019 | 0.7 | 32 – 42 |
| | NUVEL-1A | 135 | 135.1 ± 13.8 | 0.021 | 0.7 | 28 – 40 |
| | REVEL | 135 | 135.2 ± 13.8 | 0.026 | 0.8 | 32 – 43 |
| *North America-Rivera* | | | | | | |
| convergent | GSRM v2.1 | 90 | 90.5 ± 36.2 | 0.419 | 108.4 | 4 – 15 |
| | MORVEL56 | 90 | 93.1 ± 37.2 | 0.437 | 107.4 | 4 – 15 |
| | NUVEL-1A | 90 | 178.3 ± 36.3 | 0.504 | 112.0 | 5 – 14 |
| *North Bismarck-Pacific* | | | | | | |
| convergent | GSRM v2.1 | 90 | 101.2 ± 6.3 | 0.029 | 0.2 | 2 – 20 |
| | MORVEL56 | 90 | 120.4 ± 16.8 | 0.289 | 1.4 | 4 – 29 |
| *North Bismarck-Solomon Sea* | | | | | | |
| convergent | GSRM v2.1 | 90 | 81.1 ± 26.1 | 0.230 | 0.6 | 50 – 55 |
| | MORVEL56 | 90 | 78.7 ± 32.0 | 0.271 | 0.6 | 35 – 40 |
| *North Bismarck-Woodlark* | | | | | | |
| convergent | GSRM v2.1 | 90 | 73.3 ± 27.8 | 0.251 | 1.3 | 36 – 44 |

| | | | | | | |
|---|---|---|---|---|---|---|
| | MORVEL56 | 90 | 71.1 ± 31.4 | 0.243 | 0.9 | 33 – 41 |
| *Nazca-South America* | | | | | | |
| convergent | GSRM v2.1 | 90 | 94.3 ± 15.1 | 0.061 | 2.0 | 54 – 90 |
| | MORVEL56 | 90 | 97.0 ± 15.6 | 0.078 | 1.9 | 49 – 90 |
| | NUVEL-1A | 90 | 94.8 ± 15.3 | 0.064 | 2.0 | 50 – 90 |
| | REVEL | 90 | 93.9 ± 16.9 | 0.063 | 2.2 | 46 – 90 |
| *Okhotsk-Pacific* | | | | | | |
| convergent | GSRM v2.1 | 90 | 92.7 ± 12.6 | 0.207 | 0.9 | 52 – 87 |
| | MORVEL56 | 90 | 93.8 ± 12.1 | 0.193 | 1.0 | 59 – 87 |
| | REVEL | 90 | 91.3 ± 12.7 | 0.199 | 1.3 | 57 – 90 |
| *Okinawa-Philippine Sea* | | | | | | |
| convergent | GSRM v2.1 | 90 | 88.3 ± 34.3 | 0.289 | 1.3 | 17 – 30 |
| | MORVEL56 | 90 | 88.8 ± 32.2 | 0.256 | 3.0 | 10 – 28 |
| *Pacific-Philippine Sea* | | | | | | |
| convergent | GSRM v2.1 | 90 | 111.7 ± 29.1 | 0.283 | 2.3 | 6 – 30 |
| | MORVEL56 | 90 | 88.0 ± 24.4 | 0.304 | 1.2 | 5 – 30 |
| *Pacific-Solomon Sea* | | | | | | |
| convergent | GSRM v2.1 | 90 | 72.9 ± 4.0 | 0.098 | 0.1 | 33 – 34 |
| | MORVEL56 | 90 | 74.3 ± 2.9 | 0.101 | 0.1 | 34 – 34 |
| *Pacific-Tonga* | | | | | | |
| convergent | GSRM v2.1 | 90 | 87.3 ± 12.1 | 0.161 | 9.2 | 2 – 24 |
| | MORVEL56 | 90 | 86.9 ± 11.9 | 0.150 | 2.9 | 5 – 23 |
| *Pacific-Woodlark* | | | | | | |
| convergent | GSRM v2.1 | 90 | 77.1 ± 5.6 | 0.071 | 0.3 | 32 – 34 |
| | MORVEL56 | 90 | 77.6 ± 5.9 | 0.070 | 0.3 | 37 – 39 |
| *Philippine Sea-Sunda* | | | | | | |
| convergent | GSRM v2.1 | 90 | 78.6 ± 22.5 | 0.163 | 2.8 | 50 – 71 |
| | MORVEL56 | 90 | 73.8 ± 22.8 | 0.181 | 1.9 | 47 – 69 |
| *Philippine Sea-Yangtze* | | | | | | |
| convergent | GSRM v2.1 | 90 | 87.5 ± 28.9 | 0.576 | 4.2 | 36 – 52 |
| *South America-Sandwich* | | | | | | |
| convergent | GSRM v2.1 | 90 | 76.8 ± 19.9 | 0.165 | 1.2 | 24 – 31 |
| | MORVEL56 | 90 | 77.6 ± 19.8 | 0.168 | 1.2 | 26 – 32 |
| *South Bismarck-Solomon Sea* | | | | | | |
| convergent | GSRM v2.1 | 90 | 78.9 ± 11.5 | 0.227 | 0.8 | 4 – 9 |
| | MORVEL56 | 90 | 80.4 ± 18.0 | 0.253 | 0.6 | 3 – 8 |
| *Antarctica-South America* | | | | | | |
| convergent | MORVEL56 | 90 | 66.5 ± 12.5 | 0.451 | 8.1 | 25 – 43 |
| | NUVEL-1A | 90 | 67.2 ± 12.2 | 0.350 | 7.9 | 29 – 47 |
| | REVEL | 90 | 66.8 ± 9.8 | 0.540 | 7.5 | 29 – 46 |
| transform (L) | GSRM v2.1 | 45 | 40.7 ± 26.6 | 0.148 | 0.4 | 27 – 35 |
| | MORVEL56 | 45 | 42.4 ± 27.0 | 0.158 | 0.3 | 27 – 34 |
| | NUVEL-1A | 45 | 44.2 ± 30.9 | 0.181 | 0.6 | 32 – 38 |
| | REVEL | 45 | 54.5 ± 27.0 | 0.217 | 0.8 | 27 – 34 |
| *Australia-New Hebrides* | | | | | | |
| convergent | MORVEL56 | 90 | 82.4 ± 24.4 | 0.251 | 1.4 | 23 – 45 |
| *Birds Head-Banda Sea* | | | | | | |
| convergent | MORVEL56 | 90 | 46.5 ± 14.8 | 0.290 | 1.2 | 12 – 20 |
| *Birds Head-Caroline* | | | | | | |
| convergent | MORVEL56 | 90 | 102.4 ± 19.2 | 0.205 | 1.3 | 17 – 33 |
| *Birds Head-Molucca Sea* | | | | | | |
| convergent | MORVEL56 | 90 | 84.2 ± 32.7 | 0.231 | 0.3 | 8 – 13 |
| *Birds Head-Philippine Sea* | | | | | | |
| convergent | MORVEL56 | 90 | 76.7 ± 7.6 | 0.072 | 0.7 | 9 – 14 |
| *Birds Head-Sunda* | | | | | | |

| | | | | | | |
|---|---|---|---|---|---|---|
| convergent | MORVEL56 | 90 | 83.4 ± 19.7 | 0.120 | 3.0 | 44 – 55 |
| *Banda Sea-Sunda* | | | | | | |
| convergent | MORVEL56 | 90 | 88.6 ± 27.1 | 0.155 | 4.2 | 10 – 21 |
| *Banda Sea-Timor* | | | | | | |
| convergent | MORVEL56 | 90 | 119.6 ± 16.4 | 0.197 | 2.0 | 17 – 27 |
| *Caribbean-North Andes* | | | | | | |
| convergent | MORVEL56 | 90 | 12.4 ± 12.1 | 0.585 | 0.5 | 23 – 34 |
| *Caroline-Philippine Sea* | | | | | | |
| convergent | MORVEL56 | 90 | 7.8 ± 40.9 | 0.442 | 3.6 | 3 – 11 |
| *Kermadec-Pacific* | | | | | | |
| convergent | MORVEL56 | 90 | 94.2 ± 6.7 | 0.289 | 3.7 | 6 – 34 |
| *Maoke-Woodlark* | | | | | | |
| convergent | MORVEL56 | 90 | 93.9 ± 11.9 | 0.086 | 0.7 | 4 – 12 |
| *Molucca Sea-Sunda* | | | | | | |
| convergent | MORVEL56 | 90 | 3.4 ± 36.3 | 0.600 | 10.9 | 3 – 19 |
| *North Andes-Nazca* | | | | | | |
| convergent | MORVEL56 | 90 | 99.0 ± 20.5 | 0.135 | 0.7 | 46 – 55 |
| *North Andes-South America* | | | | | | |
| convergent | MORVEL56 | 90 | 108.7 ± 22.5 | 0.315 | 2.0 | 74 – 90 |
| *Solomon Sea-Woodlark* | | | | | | |
| convergent | MORVEL56 | 90 | 95.6 ± 27.5 | 0.216 | 1.4 | 84 – 89 |
| transform (R) | GSRM v2.1 | 135 | 122.6 ± 12.7 | 0.938 | 1.0 | 26 – 30 |
| | MORVEL56 | 135 | 112.0 ± 14.8 | 0.742 | 0.8 | 88 – 90 |
| *Africa-Anatolia* | | | | | | |
| convergent | REVEL | 90 | 109.1 ± 44.9 | 0.286 | 3.9 | 5 – 12 |
| *Africa-Antarctica* | | | | | | |
| divergent | GSRM v2.1 | 0 | 153.6 ± 13.7 | 0.127 | 0.5 | 68 – 86 |
| | MORVEL56 | 0 | 155.4 ± 13.3 | 0.110 | 0.5 | 57 – 76 |
| | NUVEL-1A | 0 | 140.8 ± 13.6 | 0.130 | 0.5 | 67 – 85 |
| | REVEL | 0 | 139.3 ± 16.3 | 0.151 | 0.6 | 64 – 76 |
| *Africa-Arabia* | | | | | | |
| divergent | GSRM v2.1 | 0 | 19.6 ± 22.8 | 0.092 | 0.8 | 13 – 30 |
| | MORVEL56 | 0 | 0.1 ± 13.2 | 0.040 | 2.1 | 6 – 29 |
| | NUVEL-1A | 0 | 20.8 ± 32.9 | 0.133 | 4.8 | 7 – 24 |
| | REVEL | 0 | 170.8 ± 17.0 | 0.144 | 2.1 | 2 – 22 |
| transform (L) | GSRM v2.1 | 45 | 18.0 ± 39.2 | 0.258 | 1.0 | 11 – 20 |
| | MORVEL56 | 45 | 9.7 ± 27.0 | 0.358 | 0.7 | 7 – 14 |
| | NUVEL-1A | 45 | 66.1 ± 24.6 | 0.276 | 2.1 | 6 – 17 |
| | REVEL | 45 | 178.4 ± 32.5 | 0.327 | 1.4 | 0 – 9 |
| *Africa-North America* | | | | | | |
| divergent | GSRM v2.1 | 0 | 12.8 ± 35.5 | 0.111 | 0.2 | 52 – 79 |
| | MORVEL56 | 0 | 11.8 ± 30.0 | 0.110 | 0.2 | 45 – 72 |
| | NUVEL-1A | 0 | 11.2 ± 30.1 | 0.108 | 0.2 | 46 – 74 |
| | REVEL | 0 | 15.5 ± 20.9 | 0.117 | 0.3 | 55 – 85 |
| *Africa-South America* | | | | | | |
| divergent | GSRM v2.1 | 0 | 171.3 ± 29.7 | 0.146 | 0.9 | 49 – 90 |
| | MORVEL56 | 0 | 172.6 ± 30.9 | 0.144 | 1.0 | 46 – 90 |
| | NUVEL-1A | 0 | 172.1 ± 30.8 | 0.145 | 1.0 | 47 – 90 |
| | REVEL | 0 | 172.6 ± 31.6 | 0.142 | 0.9 | 47 – 90 |
| *Africa-Somalia* | | | | | | |
| divergent | GSRM v2.1 | 0 | 43.8 ± 19.0 | 0.422 | 5.0 | 10 – 38 |
| | REVEL | 0 | 172.6 ± 29.5 | 0.229 | 3.7 | 6 – 50 |
| *Amur-Eurasia* | | | | | | |
| divergent | GSRM v2.1 | 0 | 86.0 ± 24.5 | 0.914 | 32.9 | 2 – 20 |
| | MORVEL56 | 0 | 62.8 ± 19.8 | 0.697 | 11.3 | 9 – 37 |

| | | | | | | |
|---|---|---|---|---|---|---|
| | REVEL | 0 | 0.8 ± 23.7 | 0.418 | 17.1 | 17 – 41 |
| *Amur-Okinawa* | | | | | | |
| divergent | GSRM v2.1 | 0 | 133.3 ± 30.0 | 0.205 | 2.2 | 7 – 12 |
| | MORVEL56 | 0 | 125.5 ± 25.5 | 0.189 | 1.1 | 2 – 5 |
| *Antarctica-Australia* | | | | | | |
| divergent | GSRM v2.1 | 0 | 33.2 ± 39.7 | 0.181 | 1.1 | 49 – 90 |
| | MORVEL56 | 0 | 32.1 ± 39.6 | 0.180 | 1.2 | 43 – 90 |
| | NUVEL-1A | 0 | 43.4 ± 39.8 | 0.186 | 1.2 | 46 – 90 |
| | REVEL | 0 | 33.4 ± 40.0 | 0.179 | 1.1 | 51 – 90 |
| *Antarctica-Nazca* | | | | | | |
| divergent | GSRM v2.1 | 0 | 136.0 ± 3.0 | 0.196 | 1.4 | 75 – 89 |
| | MORVEL56 | 0 | 137.1 ± 2.7 | 0.189 | 1.3 | 69 – 84 |
| | NUVEL-1A | 0 | 136.4 ± 2.8 | 0.191 | 1.3 | 74 – 88 |
| | REVEL | 0 | 131.2 ± 3.4 | 0.245 | 1.9 | 70 – 82 |
| *Antarctica-Pacific* | | | | | | |
| divergent | GSRM v2.1 | 0 | 42.8 ± 4.1 | 0.235 | 2.1 | 25 – 79 |
| | MORVEL56 | 0 | 41.9 ± 4.2 | 0.231 | 2.1 | 23 – 76 |
| | NUVEL-1A | 0 | 43.1 ± 4.2 | 0.238 | 2.1 | 25 – 79 |
| | REVEL | 0 | 44.8 ± 4.1 | 0.251 | 2.2 | 28 – 78 |
| *Antarctica-Somalia* | | | | | | |
| divergent | GSRM v2.1 | 0 | 166.4 ± 42.9 | 0.031 | 0.6 | 35 – 65 |
| | REVEL | 0 | 144.0 ± 16.0 | 0.081 | 0.7 | 40 – 65 |
| *Arabia-Somalia* | | | | | | |
| divergent | GSRM v2.1 | 0 | 170.3 ± 21.6 | 0.058 | 0.3 | 19 – 32 |
| | REVEL | 0 | 161.9 ± 13.0 | 0.096 | 0.1 | 14 – 26 |
| *Australia-Somalia* | | | | | | |
| divergent | GSRM v2.1 | 0 | 16.0 ± 43.8 | 0.165 | 2.0 | 22 – 38 |
| | REVEL | 0 | 17.8 ± 37.2 | 0.171 | 1.9 | 25 – 41 |
| *Australia-Tonga* | | | | | | |
| divergent | GSRM v2.1 | 0 | 17.5 ± 28.2 | 0.128 | 19.4 | 1 – 11 |
| | MORVEL56 | 0 | 17.6 ± 43.8 | 0.121 | 4.6 | 1 – 10 |
| *Burma-Sunda* | | | | | | |
| divergent | GSRM v2.1 | 0 | 167.8 ± 24.1 | 0.148 | 2.3 | 10 – 14 |
| | MORVEL56 | 0 | 156.5 ± 41.0 | 0.150 | 3.0 | 4 – 14 |
| *Cocos-Nazca* | | | | | | |
| divergent | GSRM v2.1 | 0 | 135.2 ± 25.9 | 0.219 | 0.9 | 35 – 58 |
| | MORVEL56 | 0 | 156.7 ± 40.4 | 0.186 | 1.0 | 40 – 62 |
| | NUVEL-1A | 0 | 136.1 ± 30.3 | 0.209 | 1.3 | 23 – 45 |
| *Cocos-Pacific* | | | | | | |
| divergent | GSRM v2.1 | 0 | 136.1 ± 44.4 | 0.218 | 1.3 | 25 – 40 |
| | MORVEL56 | 0 | 135.9 ± 42.7 | 0.218 | 1.2 | 25 – 40 |
| | NUVEL-1A | 0 | 133.4 ± 40.9 | 0.230 | 1.6 | 19 – 34 |
| *Easter-Nazca* | | | | | | |
| divergent | GSRM v2.1 | 0 | 83.0 ± 29.2 | 0.428 | 3.8 | 1 – 6 |
| | MORVEL56 | 0 | 93.2 ± 25.1 | 0.459 | 5.5 | 1 – 6 |
| *Easter-Pacific* | | | | | | |
| divergent | GSRM v2.1 | 0 | 43.1 ± 36.2 | 0.210 | 9.4 | 1 – 6 |
| | MORVEL56 | 0 | 42.9 ± 12.6 | 0.194 | 9.6 | 1 – 6 |
| *Eurasia-North America* | | | | | | |
| divergent | GSRM v2.1 | 0 | 177.8 ± 19.6 | 0.198 | 2.0 | 2 – 68 |
| | MORVEL56 | 0 | 4.2 ± 17.5 | 0.200 | 1.0 | 6 – 78 |
| | NUVEL-1A | 0 | 2.9 ± 17.2 | 0.199 | 1.4 | 2 – 78 |
| | REVEL | 0 | 4.1 ± 18.0 | 0.199 | 1.8 | 1 – 72 |
| *Eurasia-Okhotsk* | | | | | | |
| divergent | GSRM v2.1 | 0 | 95.7 ± 11.4 | 0.649 | 2.6 | 12 – 28 |

| | | | | | | |
|---|---|---|---|---|---|---|
| | MORVEL56 | 0 | 16.6 ± 11.9 | 0.138 | 2.7 | 21 – 30 |
| *Eurasia-Yangtze* | | | | | | |
| divergent | GSRM v2.1 | 0 | 68.9 ± 29.1 | 0.698 | 3.9 | 32 – 52 |
| *India-Somalia* | | | | | | |
| divergent | GSRM v2.1 | 0 | 6.2 ± 16.3 | 0.132 | 1.3 | 27 – 51 |
| | REVEL | 0 | 7.7 ± 23.8 | 0.132 | 1.1 | 36 – 59 |
| *Juan de Fuca-Pacific* | | | | | | |
| divergent | GSRM v2.1 | 0 | 157.1 ± 23.3 | 0.146 | 0.6 | 42 – 52 |
| | MORVEL56 | 0 | 157.2 ± 23.1 | 0.147 | 0.6 | 42 – 52 |
| | NUVEL-1A | 0 | 155.7 ± 21.2 | 0.146 | 0.5 | 72 – 82 |
| *Mariana-Philippine Sea* | | | | | | |
| divergent | GSRM v2.1 | 0 | 7.1 ± 24.8 | 0.197 | 4.8 | 0 – 10 |
| | MORVEL56 | 0 | 4.0 ± 16.9 | 0.084 | 1.3 | 3 – 14 |
| *Niuafo'ou-Tonga* | | | | | | |
| divergent | GSRM v2.1 | 0 | 44.7 ± 19.2 | 0.160 | 2.2 | 2 – 6 |
| | MORVEL56 | 0 | 27.1 ± 15.6 | 0.145 | 2.6 | 2 – 5 |
| *Nazca-Pacific* | | | | | | |
| divergent | GSRM v2.1 | 0 | 44.5 ± 36.5 | 0.203 | 0.9 | 55 – 90 |
| | MORVEL56 | 0 | 43.5 ± 37.9 | 0.198 | 0.9 | 54 – 90 |
| | NUVEL-1A | 0 | 44.5 ± 36.4 | 0.205 | 1.0 | 53 – 89 |
| | REVEL | 0 | 43.2 ± 40.4 | 0.208 | 1.1 | 53 – 90 |
| *Okinawa-Yangtze* | | | | | | |
| divergent | GSRM v2.1 | 0 | 9.4 ± 25.2 | 0.185 | 1.1 | 7 – 20 |
| *Scotia-Sandwich* | | | | | | |
| divergent | GSRM v2.1 | 0 | 30.7 ± 12.6 | 0.136 | 1.0 | 22 – 28 |
| | MORVEL56 | 0 | 29.0 ± 13.0 | 0.134 | 0.9 | 22 – 28 |
| *Aegean Sea-Anatolia* | | | | | | |
| divergent | MORVEL56 | 0 | 74.7 ± 36.7 | 0.335 | 1.4 | 4 – 11 |
| *Australia-Conway Reef* | | | | | | |
| divergent | MORVEL56 | 0 | 18.9 ± 20.8 | 0.090 | 1.6 | 1 – 5 |
| *Australia-Kermadec* | | | | | | |
| divergent | MORVEL56 | 0 | 7.3 ± 12.6 | 0.088 | 3.1 | 1 – 17 |
| *Balmoral Reef-Pacific* | | | | | | |
| divergent | MORVEL56 | 0 | 49.9 ± 21.1 | 0.214 | 0.7 | 87 – 90 |
| *Conway Reef-New Hebrides* | | | | | | |
| divergent | MORVEL56 | 0 | 3.0 ± 16.8 | 0.135 | 2.7 | 3 – 10 |
| *Caribbean-North America* | | | | | | |
| transform (L) | GSRM v2.1 | 45 | 50.7 ± 15.7 | 0.032 | 1.2 | 67 – 80 |
| | MORVEL56 | 45 | 49.7 ± 17.1 | 0.032 | 1.2 | 61 – 74 |
| | NUVEL-1A | 45 | 46.2 ± 15.2 | 0.053 | 1.3 | 76 – 87 |
| | REVEL | 45 | 46.0 ± 10.1 | 0.061 | 2.1 | 63 – 75 |
| *Caroline-Pacific* | | | | | | |
| transform (L) | GSRM v2.1 | 45 | 18.3 ± 35.5 | 0.223 | 2.0 | 16 – 24 |
| | MORVEL56 | 45 | 103.9 ± 15.3 | 0.423 | 3.0 | 4 – 16 |
| *North Bismarck-South Bismarck* | | | | | | |
| transform (L) | GSRM v2.1 | 45 | 69.4 ± 43.5 | 0.865 | 0.3 | 8 – 11 |
| | MORVEL56 | 45 | 69.0 ± 44.1 | 0.935 | 0.3 | 8 – 10 |
| transform (R) | GSRM v2.1 | 135 | 43.9 ± 39.8 | 0.519 | 0.9 | 4 – 9 |
| | MORVEL56 | 135 | 48.9 ± 29.5 | 0.602 | 0.5 | 4 – 9 |
| *Niuafo'ou-Pacific* | | | | | | |
| transform (L) | GSRM v2.1 | 45 | 43.1 ± 14.2 | 0.130 | 0.7 | 22 – 26 |
| | MORVEL56 | 45 | 45.8 ± 14.0 | 0.126 | 0.7 | 22 – 25 |
| *South America-Scotia* | | | | | | |
| transform (L) | GSRM v2.1 | 45 | 5.4 ± 23.1 | 0.317 | 0.8 | 53 – 62 |
| | MORVEL56 | 45 | 63.4 ± 38.0 | 0.262 | 0.7 | 47 – 57 |

| | | | | | | |
|---|---|---|---|---|---|---|
| | NUVEL-1A | 45 | 78.1 ± 41.7 | 0.286 | 1.6 | 37 – 48 |
| *Arabia-Anatolia* | | | | | | |
| transform (L) | MORVEL56 | 45 | 55.8 ± 18.7 | 0.181 | 0.9 | 7 – 14 |
| | REVEL | 45 | 53.9 ± 19.6 | 0.175 | 1.1 | 10 – 16 |
| *Australia-Birds Head* | | | | | | |
| transform (L) | MORVEL56 | 45 | 34.5 ± 7.0 | 0.085 | 0.4 | 51 – 53 |
| *Australia-Balmoral Reef* | | | | | | |
| transform (L) | MORVEL56 | 45 | 55.1 ± 8.6 | 0.040 | 0.6 | 35 – 40 |
| *Australia-Futuna* | | | | | | |
| transform (L) | MORVEL56 | 45 | 31.0 ± 26.6 | 0.152 | 0.3 | 2 – 4 |
| *Australia-Maoke* | | | | | | |
| transform (L) | MORVEL56 | 45 | 13.7 ± 25.0 | 0.218 | 0.2 | 10 – 11 |
| *Birds Head-Maoke* | | | | | | |
| transform (L) | MORVEL56 | 45 | 49.6 ± 24.0 | 0.121 | 0.6 | 87 – 90 |
| *Balmoral Reef-Conway Reef* | | | | | | |
| transform (L) | MORVEL56 | 45 | 88.7 ± 34.2 | 0.269 | 1.3 | 3 – 6 |
| *Banda Sea-Molucca Sea* | | | | | | |
| transform (L) | MORVEL56 | 45 | 88.2 ± 43.4 | 0.313 | 1.3 | 0 – 9 |
| transform (R) | MORVEL56 | 135 | 87.7 ± 25.2 | 0.315 | 1.5 | 1 – 9 |
| *Futuna-Pacific* | | | | | | |
| transform (L) | MORVEL56 | 45 | 89.7 ± 22.3 | 0.296 | 0.2 | 5 – 6 |
| *Manus-North Bismarck* | | | | | | |
| transform (L) | MORVEL56 | 45 | 164.7 ± 33.4 | 0.412 | 0.2 | 0 – 2 |
| *Antarctica-Sandwich* | | | | | | |
| transform (R) | GSRM v2.1 | 135 | 117.3 ± 8.7 | 0.171 | 0.7 | 22 – 24 |
| | MORVEL56 | 135 | 121.4 ± 11.9 | 0.167 | 0.6 | 21 – 23 |
| *Arabia-India* | | | | | | |
| transform (R) | GSRM v2.1 | 135 | 82.6 ± 39.7 | 0.387 | 0.6 | 4 – 12 |
| | MORVEL56 | 135 | 167.4 ± 42.4 | 0.243 | 2.1 | 58 – 67 |
| | NUVEL-1A | 135 | 164.1 ± 40.9 | 0.230 | 2.1 | 34 – 41 |
| *Aegean Sea-Eurasia* | | | | | | |
| transform (R) | GSRM v2.1 | 135 | 133.7 ± 21.9 | 0.121 | 1.7 | 60 – 69 |
| | MORVEL56 | 135 | 129.4 ± 29.6 | 0.185 | 2.0 | 79 – 89 |
| *Australia-Niuafo'ou* | | | | | | |
| transform (R) | GSRM v2.1 | 135 | 144.1 ± 10.7 | 0.121 | 0.8 | 10 – 16 |
| | MORVEL56 | 135 | 145.6 ± 10.8 | 0.123 | 0.5 | 10 – 14 |
| *Pacific-Rivera* | | | | | | |
| transform (R) | GSRM v2.1 | 135 | 137.9 ± 3.4 | 0.113 | 0.2 | 5 – 8 |
| | MORVEL56 | 135 | 137.9 ± 3.4 | 0.113 | 0.2 | 5 – 8 |
| | NUVEL-1A | 135 | 137.5 ± 4.3 | 0.056 | 0.4 | 10 – 14 |
| *Anatolia-Eurasia* | | | | | | |
| transform (R) | MORVEL56 | 135 | 125.6 ± 27.0 | 0.188 | 1.6 | 6 – 16 |
| | REVEL | 135 | 130.7 ± 23.1 | 0.155 | 1.2 | 11 – 18 |
| *Manus-South Bismarck* | | | | | | |
| transform (R) | MORVEL56 | 135 | 169.8 ± 26.8 | 0.234 | 1.1 | 1 – 4 |
| *North Andes-Panama* | | | | | | |
| transform (R) | MORVEL56 | 135 | 103.1 ± 35.5 | 0.188 | 0.9 | 38 – 43 |
| *Sunda-Timor* | | | | | | |
| transform (R) | MORVEL56 | 135 | 100.7 ± 4.7 | 0.207 | 2.2 | 7 – 14 |
